# Semantic–Electromagnetic Inversion With Pretrained Multimodal Generative Model

**DOI:** 10.1002/advs.202406793

**Published:** 2024-09-09

**Authors:** Yanjin Chen, Hongrui Zhang, Jie Ma, Tie Jun Cui, Philipp del Hougne, Lianlin Li

**Affiliations:** ^1^ State Key Laboratory of Advanced Optical Communication Systems and Networks School of Electronics Peking University Beijing 100871 China; ^2^ State Key Laboratory of Millimeter Waves Southeast University Nanjing 210096 China; ^3^ Pazhou Laboratory (Huangpu) Guangzhou 510555 China; ^4^ Université Rennes CNRS IETR – UMR 6164 Rennes F‐35000 France

**Keywords:** inverse scattering, microwave imaging, pretrained large‐capacity foundation models, semantic–electromagnetic inverse problem

## Abstract

Across diverse domains of science and technology, electromagnetic (EM) inversion problems benefit from the ability to account for multimodal prior information to regularize their inherent ill‐posedness. Indeed, besides priors that are formulated mathematically or learned from quantitative data, valuable prior information may be available in the form of text or images. Besides handling semantic multimodality, it is furthermore important to minimize the cost of adapting to a new physical measurement operator and to limit the requirements for costly labeled data. Here, these challenges are tackled with a frugal and multimodal semantic–EM inversion technique. The key ingredient is a multimodal generator of reconstruction results that can be pretrained, being agnostic to the physical measurement operator. The generator is fed by a multimodal foundation model encoding the multimodal semantic prior and a physical adapter encoding the measured data. For a new physical setting, only the lightweight physical adapter is retrained. The authors’ architecture also enables a flexible iterative step‐by‐step solution to the inverse problem where each step can be semantically controlled. The feasibility and benefits of this methodology are demonstrated for three EM inverse problems: a canonical two‐dimensional inverse‐scattering problem in numerics, as well as three‐dimensional and four‐dimensional compressive microwave meta‐imaging experiments.

## Graphical abstract

This work presents a semantic–EM inversion method capable of incorporating multimodal semantic priors in a flexible and frugal manner. It shows great advantages in handling semantic multimodality through a semantic‐guided step‐by‐step manner and minimizing the cost of adapting to a new physical measurement operator and to limit the requirements for costly labeled training data.

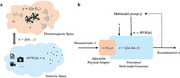

## 1 Introduction

An electromagnetic (EM) wavefront is scattered upon interacting with matter. Based on the scattered wavefront, EM inversion aims at inferring properties of the incident wavefront or properties of the matter (e.g., shape, material composition, etc.). Prominent applications involving EM inversion include diverse types of computational imaging in applications such as non‐destructive testing,^[^ ^1^ ^]^ medical diagnosis,^[^ ^2^, ^3^, ^4^, ^5^ ^]^ subsurface exploration,^[^ ^6^, ^7^, ^8^, ^9^, ^10^ ^]^ security screening,^[^ ^11^, ^12^ ^]^ or cometary science.^[^ ^13^ ^]^ Such inverse problems (aiming to deduce the “cause” of an observed “effect”) are notoriously ill‐posed because the observed information is insufficient to unambiguously retrieve the sought‐after unknowns (multiple “causes” could explain the observed “effect”). In light of Bayes’ rule,^[^ ^14^ ^]^ the ill‐posedness can be addressed by incorporating prior knowledge about the unknowns in the solution process. Traditionally, prior knowledge is formulated mathematically (e.g., sparsity of the unknowns), a physical forward model is known, and an iterative algorithm identifies a solution that best explains the measurements under the constraints imposed by the prior. The proliferation of deep learning has enabled a plethora of new inversion techniques.^[^ ^15^, ^16^ ^]^ First, if no physical forward model exists, a forward model of the measurement process can be learned in a supervised manner based on matched input–output pairs. Second, a direct end‐to‐end inverse mapping from measurements to reconstruction can be learned.^[^ ^17^ ^]^ In this case, no explicit physical or learned forward model is required but the reconstruction network is very specific to a given physical setting, implying a high sensitivity to perturbations and a lack of adaptability to different settings. Third, the prior can be learned from quantitative training data. One surprisingly simple but effective such approach consists in using networks trained for artifact removal or denoising (i.e., without any specificity to the considered setting);^[^ ^18^ ^]^ iterative optimization procedures (which can be interpreted as unrolled networks^[^ ^19^ ^]^) can alternate between enforcing data fidelity and applying such a network. Another important instance of learned priors consists in training a generative model which implicitly captures the “natural” distribution of the unknowns;^[^ ^20^, ^21^ ^]^ the generative model maps a random input to a typical realization of the unknowns and can be trained in an unsupervised manner (e.g., as variational autoencoder or generative adversarial network); in combination with a forward model, the input to the generative model can then be optimized by gradient descent to minimize the data misfit, while the generative model enforces its prior. Noteworthy are further training‐free methods leveraging a “deep image prior” (DIP) by iteratively optimizing the weights of an untrained generative model in combination with a known forward model;^[^ ^22^, ^23^, ^24^, ^25^, ^26^ ^]^ DIPs thereby leverage an architectural bias of the generative model but the underlying theory remains poorly understood.^[^ ^16^ ^]^

Applications of the above‐surveyed deep‐learning‐based techniques to EM inversion in contexts such as computational imaging have, to date, generally been limited to learning a prior from quantitative training data comprising output examples or matched input–output pairs. Yet, important prior information is often formulated in natural language when it originates from human reasoning or recognition. Recent releases of large‐language foundation models (LFMs) have paved the way to making use of such semantic priors in EM inversion. Very recently,^[^ ^27^ ^]^ an LFM was leveraged to regularize EM inversion based on semantic priors formulated in natural language. Yet, it^[^ ^27^ ^]^ did not address the challenge of frugally and flexibly adapting to different physical measurement operators. In addition, it^[^ ^27^ ^]^ was limited to semantic priors in natural language whereas important application scenarios involve multimodal priors such as combinations of a text and an image of some kind. The image prior could be a human‐drawn sketch, a contour, a result from a related inversion in the case of multiphysics problems, and so on. Naturally, the ability to handle such multimodal priors also lends itself to implementing iterative step‐by‐step solutions, where the reconstruction from the previous step can be used as a prior in the form of an image, possibly controlled by text in natural language.

We hypothesize that an approach to semantic‐EM inversion based on a multimodal generative model (MGM) can largely address the above‐raised concerns. The MGM is trained to implicitly capture the “natural” distribution of the unknowns, irrespective of the specific physical measurement setup. Training the MGM requires matched triplets of a realization of the unknown and corresponding text and image priors, but no corresponding EM measurements. Hence, the MGM can be pretrained and used in combination with different lightweight physical adapters that are specific to a given physical measurement operator; given the pretrained MGM, the physical adapters are trained in a supervised manner based on quadruplets of realizations of the unknown, corresponding text and image priors, and corresponding EM measurements. Knowledge of the realizations of the unknown is dispensable if the physical forward model is known. Generative foundation models have rapidly developed in recent years in contexts such as the generation of human‐level images,^[^ ^28^, ^29^ ^]^ texts,^[^ ^30^, ^31^ ^]^ music,^[^ ^32^, ^33^ ^]^ and videos;^[^ ^34^, ^35^ ^]^ for instance, ChatGPT^[^ ^36^ ^]^ for multimodality interactions, StableDiffusion^[^ ^37^ ^]^ for text‐guided image generation, Sora^[^ ^38^ ^]^ for text‐guided video generation, or Suno^[^ ^39^ ^]^ for music generation. Recent advancements hint at the tremendous potential of generative models in diverse disciplines, for instance, for the discovery of new protein structures with an intended biological activity,^[^ ^40^ ^]^ potential new drug molecules,^[^ ^41^ ^]^ or novel materials with superior properties.^[^ ^42^ ^]^

In this work, we demonstrate the semantic–EM inversion technique based on a pretrained system‐agnostic MGM and a lightweight physical adapter characterizing the measurement system. Our technique can handle multimodal priors involving semantics and images; it facilitates operation with multiple measurement systems, and it can flexibly be deployed in a untrained or trained manner. We first apply our technique to a canonical 2D EM inverse‐scattering problem. Then, we apply our technique experimentally to the 3D and 4D meta‐imaging of human postures and motion in indoor settings. Meta‐imaging is a computational imaging technique leveraging low‐cost programmable metasurfaces to flexibly define the physical measurement operator.^[^ ^43^ ^]^ In recent years, metasurfaces have achieved remarkable results in the field of imaging, for instance, learned metasurfaces have significantly improved depth estimation accuracy in 3D imaging.^[^ ^44^ ^]^ These programmable metasurfaces are widely envisioned to be part of next‐generation wireless networks that integrate communications and sensing capabilities. Overall, we expect our presented semantic‐EM inversion strategy to pave the way to a flexible and frugal use of multimodal semantic prior information in EM inversion.

## 2 Results

### 2.1 Problem Statement

**Figure 1:**
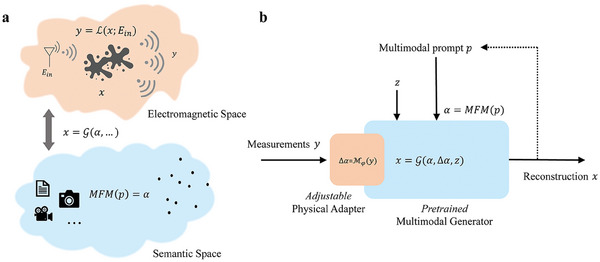
Principle of our proposed semantic–EM inversion technique with a pretrained multimodal generative model (MGM). a) Illustration of the electromagnetic and semantic spaces and their relation. The semantic space is characterized by a multimodal foundation model α  =  *MFM*(p), where p is the multimodal semantic prompt comprising text, image, and so on. An MGM maps the encoded multimodal semantic prior α, as well as additional inputs, to the sought‐after unknown *x* in the physical world: x=G(α,Δα,z). The measurement model in the electromagnetic space is characterized by $y\;=\;L(x;E_{in})$, where L is governed by Maxwell's equations. b) Our semantic‐EM inversion framework is composed of a pretrained MGM and a physical adapter. Measurements are fed via the physical adapter $M_{\varphi }$ into the MGM. In addition, the MGM takes the multimodal prior encoded by the MFM and a random vector *z* as inputs. If desired, the MGM can be deployed in an iterative step‐by‐step manner by feeding the reconstruction from a previous step back via the multimodal prior prompt (indicated by the dotted line).

EM inversion aims at inferring a meaningful reconstruction of a high‐dimensional unknown $x\in R^{m}$ that can explain the low‐dimensional observation $y\in R^{n}$, where *n* < *m*. Here, *m* and *n* represent the dimensions of the unknown *x* and the observation *y*, respectively. This inverse problem is generally ill‐posed but could become well‐posed if we can supply adequate prior information about the unknown. In this paper, we explore a technique to frugally and flexibly make use of multimodal semantic prior information to regularize the EM inversion. Our considered semantic‐EM problem is illustrated in **Figure** **1** and can be formalized as follows:

(1) $$y=L\left(x;E_{\mathrm{in}}\right)$$

(2) $$x=G\left(\alpha ,\Delta \alpha ,z\right)$$

and

(3) $$\alpha =MFM\left(p\right)$$

The physical measurement operator L defined in Equation (1) maps the sought‐after unknown *x* (e.g., a dielectric constant distribution) to the EM measurements *y*; *E*_in_ denotes the incident wavefront probing the scene. The “natural” distribution of realizations of the unknown *x* is captured by the MGM G which maps a triplet (α, Δα, *z*) to a realization of *x*, as seen in Equation (2). Here, *p* is the multimodal semantic prior. As seen in Equation (3), the multimodal foundation model (MFM) encodes a multimodal semantic prior *p* in the semantic embedding α which carries high‐level information about the unknown. The multimodal semantic prior *p* can be a combination of text and image, as seen below. Δα is an embedding of low‐level information about the unknown. Specifically, a physical adapter network $M_{\varphi }$ embeds the EM measurements *y* in Δα. The third input of the MGM, *z*, is a vector of random numbers to account for the stochastic nature of the MGM. The operation of our semantic‐EM inversion technique can take multiple forms. On the one hand, it can be employed non‐iteratively. EM measurements are fed into the physical adapter, multimodal semantic prompts are fed into the MFM, and the MGM maps the encoded results to a reconstruction of the unknown. However, an iterative step‐by‐step solution is also possible whereby the reconstruction from a previous step can be fed as part of the multimodal semantic prior into the MFM (indicated by the dotted lines in Figure 1b). More details about our framework can be found in Note S1, Supporting Information.

Recent releases of pretrained MFMs enable us to use a frozen pretrained MFM without having to train it ourselves. Moreover, we can pretrain our MGM based on matched training examples (*x*, *p*) irrespective of the physical measurement operator. Note that in order to train the MGM, there is hence no need to know the EM measurements *y* corresponding to these (*x*, *p*). Once the MGM is pretrained, we freeze it. Now, we are left with identifying the physical adapter $M_{\varphi }$ which is specific to the physical measurement operator. In case the physical forward model L is known, we can obtain the parameter φ of the physical adapter via the following optimization problem:

(4) $$\hat{\varphi }=\arg\min_{\varphi }∥\;y-L\left(G\left(MFM\left(p\right),M_{\varphi }\left(y\right),z\right);E_{\mathrm{in}}\right)∥\;$$

One can readily obtain the solution $x=G(MFM\left(p\right),M_{\varphi }\left(y\right),z)$ once the value of φ is determined by solving Equation (4). Note that such inversion scheme works in an untrained way, which means we do not need some paired data to train the physical adapter. Instead, we simply input the single EM measurement *y* to be processed into the physical adapter. Through the interaction between the physical adapter $M_{\varphi }$, MGM, and the physical forward model L, we optimize the network parameters φ to ultimately obtain a feasible solution that satisfies the EM measurement *y*. Therefore, we refer to this scheme as the physics‐driven scheme. In case we do not have knowledge of the physical forward model L, we do require matched training examples (*x*, *p*, *y*) in order to learn in a supervised learning manner the weights φ of the physical adapter via the following optimization problem:

(5) $$\hat{\varphi }=\arg\min_{\varphi }E_{x,y,p,z}\left[∥\;x-G\left(MFM\left(p\right),M_{\varphi }\left(y\right),z\right)∥\right]\;$$

We refer to this scheme as the data‐driven scheme. As the MGM is already pretrained and frozen, the size of the matched training examples (*x*, *p*, *y*) to optimize the physical adapter for a given physical setting can usually be fairly small, as seen below.

### 2.2 Numerical Results of 2D Inverse Scattering

**Figure 2:**
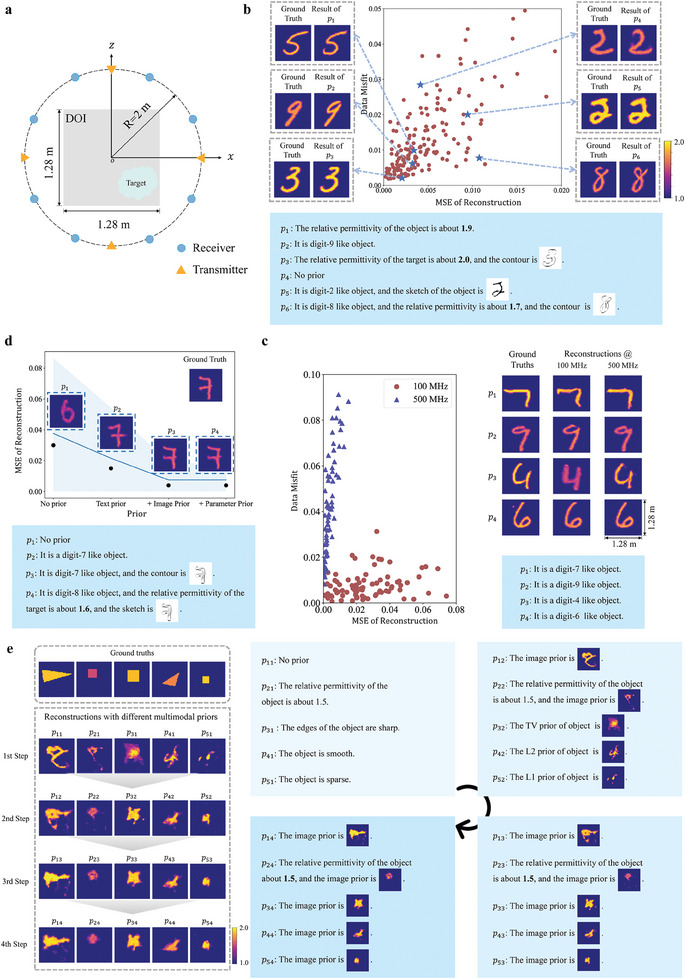
Numerical results for a canonical 2D inverse‐scattering problem. a) The considered inverse‐scattering configuration. b) Scatterplot of reconstruction MSE versus data misfit for reconstruction results. The MSE measures the accuracy with which the target has been reconstructed, and the data misfit quantifies how closely the measurements obtained with the reconstructed target match the initially measured microwaves. Six selected reconstruction results with different prompts are shown additionally. c) For two different physical measurement operators (specifically, two distinct operating frequencies of 100 and 500 MHz), distinct physical adapters are trained but the same MGM is used. A scatterplot compares the reconstruction performance in terms of MSE versus data misfit. In addition, selected reconstruction examples are shown. d) The effect of different multimodal priors on the reconstruction MSE is illustrated with four representative results. Here, the light blue shade indicates the standard deviation of the MSE and the blue line represents the average. e) Results of a semantic‐guided step‐by‐step reconstruction of out‐of‐distribution targets based on multimodal prompts incorporating reconstruction results from previous steps.

We begin by investigating the performance of our semantic‐EM inversion strategy numerically for a canonical 2D inverse‐scattering problem. As illustrated in **Figure** **2**a, the primary objective of inverse scattering is to retrieve the physical properties of the object inside the domain of interest (DoI) from the measurements of resultant scattered fields, given the known EM excitations.^[^ ^45^ ^]^ In our numerical study, the Conjugate Gradient‐Fast Fourier Transform^[^ ^46^ ^]^ full‐wave solver of Maxwell's equations is utilized to generate the simulated data, as detailed in the Methods and Note S2, Supporting Information.

We pretrain an MGM based on the existing generative adversarial network (GAN).^[^ ^47^ ^]^ The training set includes 50 000 digit‐like scatterers. The text priors are automatically generated by script, and the image priors are automatically produced using methods such as edge detection network and sketch generation network. The text and image priors are injected through the text encoder of Contrastive Language‐Image Pre‐training (CLIP)^[^ ^48^ ^]^ and image encoder, respectively, as detailed in Note S3, Supporting Information. Once the MGM is pretrained, we freeze it and turn our attention to training the physical adapter. As in the considered problem the physical measurement operator is known in closed form, we here use Equation (4) to find the weights of a lightweight physical adapter $M_{\varphi }$. It should be emphasized that this process does not involve the traditional training process. Instead, we directly use the microwave *y* to be reconstructed and the corresponding multimodal prior *p* to solve for the physical adapter $M_{\varphi }$, thereby obtaining the unknown target $x\;=\;G(MFM\left(p\right),M_{\varphi }\left(y\right),z)$. As the measurement model L is differentiable, we conveniently backpropagate the error of the objective function defined in Equation (4) to the network parameters φ using the automatic differentiation mechanism in PyTorch. Additional details are provided in Note S4, Supporting Information.

Now, we examine our technique's inverse‐scattering performance; selected results are plotted in the space of reconstruction mean square error (MSE) versus data misfits in Figure 2b, where the operational frequency is 300 MHz, and the signal to noise ratio is 20 dB. The data misfit is quantified as $∥y-L\left(G\left(MFM\left(p\right),M_{\varphi }\left(y\right),z\right);E_{\mathrm{in}}\right){∥}_{2}/∥y{∥}_{2}$. We observe that our method is capable of reconstructing the object well under various multimodal priors; meanwhile, the data misfit between the ground‐truth and predicted microwave measurement is less than 0.02. As highlighted above, the pretrained MGM universally applies to different measurement systems in the physical space because it is decoupled from the latter and characterizes the object space alone (i.e., irrespective of the measurement procedure). To illustrate this important feature of our technique, we have applied our pretrained MGM to two different measurement systems which differ with respect to the operating frequency (100 and 500 MHz). As shown in Figure 2c, overall, the reconstructions for both of them are satisfactory, testifying to the generality of the MGM; however, the reconstruction accuracy at 100 MHz is lower than that at 500 MHz, which makes sense because the resolution achievable at 100 MHz is lower than that at 500 MHz due to the longer wavelength. Meanwhile, we observe that the data misfit at 100 MHz is smaller than that at 500 MHz.

Next, in order to examine the multimodal prior's positive effect on the inversion quality, we conduct another set of numerical experiments that is documented in Figure 2d. We observe that the inversion quality can be stably improved by imposing an increasingly strong multimodal semantic prior. In particular, the microwave reconstruction of a 7‐like object is undistinguishable from a 6‐like object when the prior is absent. However, when the prior “It is a digit‐7 like object” is imposed, the inversion approaches the ground truth. Further, the resultant reconstruction is almost perfect when the contour of the target is provided as a prior. After that, there is no obvious improvement of the inversion quality even if the additional prior “The relative permittivity of the object is 1.5” is imposed. This implies that the image prior can sufficiently mitigate the ill‐posedness of the inverse scattering problem.

So far, we have only used our technique in a one‐step manner. However, our technique's ability to deal with multimodal semantic prompts allows us also to conceive an interesting iterative step‐by‐step inversion technique. A machine or human operator can incorporate the reconstruction from a previous step into the multimodal prior of the next step, possibly in combination with instructions in natural language, until some stopping criterion is reached. Selected results obtained with such an approach on out‐of‐distribution targets (geometric shapes) are presented in Figure 2e. As these targets are not digits, in the first step we can only use some universal multimodal priors, such as “The target is sparse” or we can indicate the relative permittivity of the target. Such information‐limited prompts yield an initial reconstruction result of limited quality which becomes part of the prior of the next iteration. Thereby, the preliminary result is updated in a semantic‐guided step‐by‐step way. As shown in the first column, this strategy appears effective also in cases where no prior information about the target is provided. Moreover, the results in Figure 2e demonstrate that our technique generalizes well to out‐of‐distribution targets thanks to its ability to operate in a semantic‐guided step‐by‐step manner. Although it is difficult to reconstruct the out‐of‐distribution target in the first step, eventually the step‐by‐step approach yields a reconstruction of acceptable quality. Note that human operators can guide the inversion at every step by modifying or fine‐tuning the reconstruction with commands in natural language. As detailed in Note S5, Supporting Information, we also compare our method with the DIP‐like methods, demonstrating the superiority of our method's multimodal priors in alleviating the ill‐posed nature of inverse problems.

### 2.3 Experimental Results of 3D Microwave Meta‐Imaging

We now move on to evaluating our inversion technique experimentally in the context of meta‐imaging of human postures in indoor settings. Meta‐imaging leverages a programmable metasurface to craft the physical measurement operator.^[^ ^43^ ^]^ Specifically, the scene information is multiplexed across different configurations of the programmable metasurface (an array of meta‐atoms with individually reconfigurable scattering properties) onto a single or few detectors. The simplest approach consists in measuring the field at the detector for a series of random metasurface configurations.^[^ ^49^ ^]^ We follow this compressive meta‐imaging approach in the present work, using two programmable metasurfaces operating around 2.4 and 5.5 GHz (see the Experimental Section; Note S6, Supporting Information). We note; however, that the freedom to design the physical measurement operator can in principle be leveraged to optimize the measurement procedure with respect to a specific scene^[^ ^50^, ^51^ ^]^ or end‐to‐end with respect to a specific sensing task^[^ ^52^, ^53^ ^]^ or measurement noise.^[^ ^54^ ^]^

As currently no suitable MGM is publicly available for the considered problem, we need to pretrain one. To this end, we construct a diffusion‐model‐based network (see Note S7, Supporting Information for details), where the text prior is injected into the network through the text encoder of CLIP, and the drawing prior is imposed through an off‐line trained encoder. We train the MGM based on 10 000 samples of human postures collected with a stereo optical camera; the postures are formatted as point clouds. The text priors are manually labeled, while the drawing prior is automatically labeled. Note that no microwave data is involved in training the MGM. In addition, to be able to control the importance of the multimodal prior, we use the classifier‐free guidance (CFG) technique^[^ ^55^ ^]^ during training, as detailed in the Experimental Section; Note S7, Supporting Information. Owing to the complicated indoor setting involving complex multipath propagation, we do not have access to a closed‐form of the physical measurement operator L for this meta‐imaging inversion problem. Hence, we must train the physical adapter $M_{\varphi }$ using the approach from Equation (5). We optimize its weights φ in a supervised learning procedure based on only 500 matched training examples (*x*, *p*, *y*). 18 random metasurface configurations are employed for each measurement. Additional details are provided in Note S8, Supporting Information.

**Figure 3:**
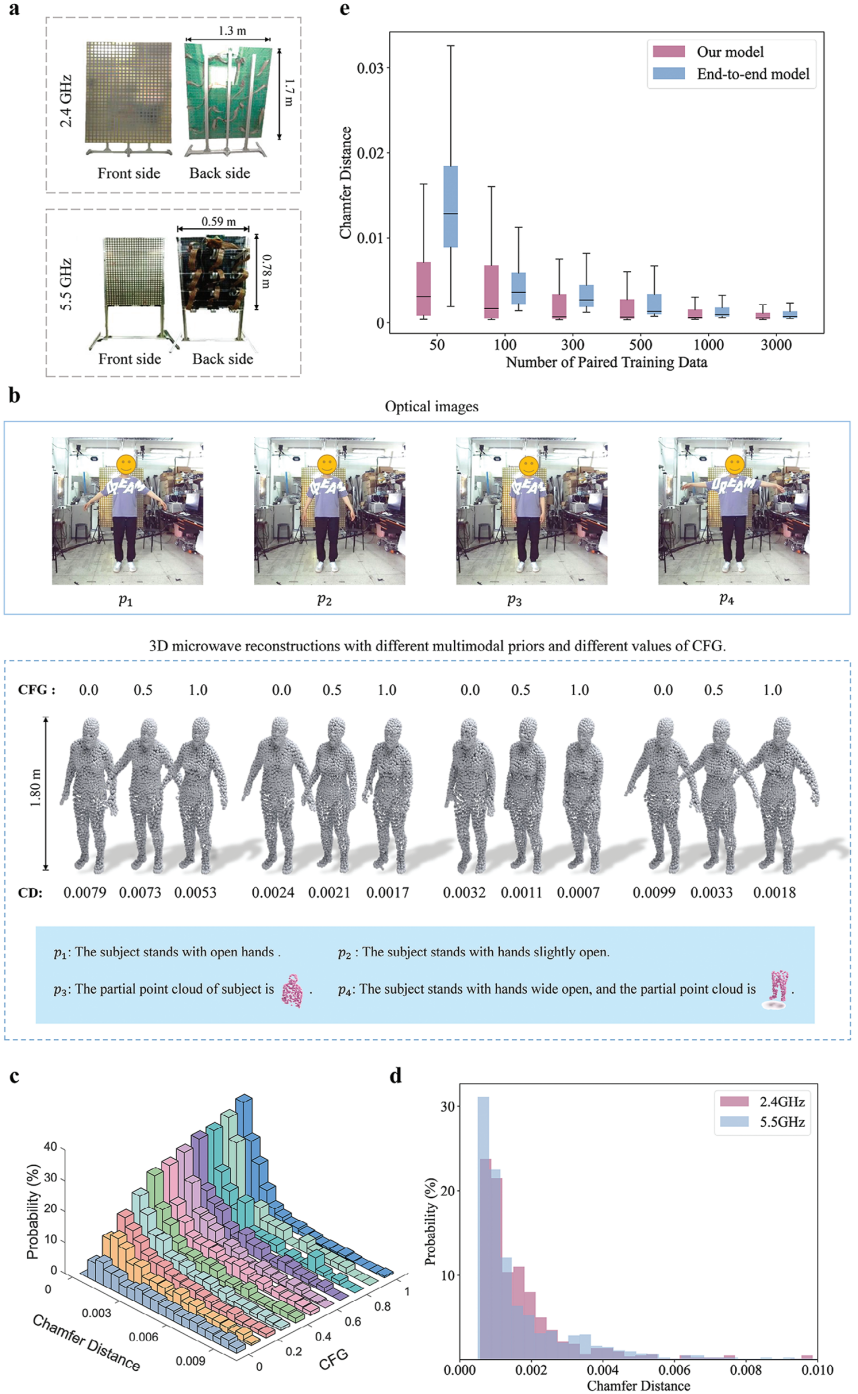
Selected experimental results for 3D microwave meta‐imaging. a) The front‐view and back‐view of the 2.4 and 5.5 GHz programmable metasurfaces. b) Selected 3D reconstruction results obtained with the 2.4 GHz system for four different priors (*p*_1_,*p*_2_,*p*_3_,*p*_4_) and different values of CFG. “CD” stands for the Chamfer distance. The optical images and the reconstruction CD are also provided at the top and bottom, respectively. c) 3D CD histogram of the reconstruction results with different values of CFG, where the 2.4 GHz system is utilized. d) CD histogram of the reconstruction results for the 2.4 and 5.5 GHz systems. e) Boxplot of the CD for 1000 test samples, comparing our method and the end‐to‐end reconstruction method after training with different amounts of paired training data.

Our first set of experiments examines the importance of the multimodal semantic prior in controlling the inversion quality. Selected results obtained with the 2.4 GHz system for varying values of CFG are displayed in **Figure** **3**b, where the value of CFG ranging from 0, 0.5, and 1 is utilized. Note that CFG = 1 means that the prior information is fully effective, CFG = 0.5 means that the prior information is partially effective, and CFG = 0.0 means that the prior information has no effect. To evaluate the reconstruction quality, we use a metric known as “Chamfer distance”^[^ ^56^ ^]^ to evaluate the similarity between two point clouds; specifically, the Chamfer distance is $d\;=\sum_{c\in C}\min_{\hat{c}\in \hat{C}}∥c-\hat{c}{∥}^{2}\;+\sum_{\hat{c}\in \hat{C}}\min_{c\in C}∥\hat{c}-c{∥}^{2}$; $\hat{C}$ and C represent the reconstructed point cloud and real point cloud, respectively; $\hat{c}$ and *c* denote points within the reconstructed and real point cloud, respectively. It can be seen from Figure 3b that the Chamfer distance of the microwave reconstruction decreases as the CFG increases. Moreover, we report a quantitative statistical analysis of the Chamfer distance over 800 testing samples for different CFG values in Figure 3c. Clearly, the inversion quality is stably improved with the growth of CFG value. This does make sense because the higher the value of CFG is, the more the prior is imposed to complement the inadequate and noisy microwave measurements. For instance, the prior “The subject stands with open hands” gives a noticeably greater spread of the hands in the reconstructions, which is closer to the ground‐truth posture of the subject. It is appealing in practical applications to control the importance of the prior in the reconstruction process. For instance, we can increase the prior's importance when we are confident in it, and decease it otherwise. This allows for a balanced integration of microwave measurements and multimodal semantic prior, enabling a higher‐quality inversion. More specifically, the more the efficient prior is imposed, the higher the reconstruction achievable accuracy is. For example, compared to the prior *p*_1_, the prior *p*_2_ offers a more informative description of the subject, resulting in a higher accuracy at the same CFG, and the priors *p*_3_ and *p*_4_ provide more priors, that is, a point cloud of parts of the subject's body, which leads to the smallest Chamfer distances.

Next, we experimentally examine the generalization capability of the pretrained MGM across the two aforementioned operational systems (2.4 and 5.5 GHz), where distinct physical adapters are trained for each measurement systems, while the pretrained MGM is fixed. Figure 3d illustrates the statistical histogram of Chamfer distances over 800 testing samples for the 2.4 and 5.5 GHz systems. Apparently, the pretrained generative model is capable of generating satisfactory results of human posture reconstructions, regardless of the utilized operational systems, and nearly identical reconstruction performance can be achieved in both systems. From these results, it can be observed that the compatibility of the pretrained MGM with different measurement systems is high. We show some selected results in Note S9, Supporting Information.

Finally, we compare our inversion strategy with a traditional end‐to‐end reconstruction network for the 5.5 GHz system. In our comparative analysis, the structure of the end‐to‐end reconstruction network is similar to that of the physical adapter, but the total number of trainable parameters is almost the same as that of the physical adapter plus the pretrained MGM. In particular, the end‐to‐end reconstruction network involves up to 14 million parameters in training, while our physical adapter only involves 3 million parameters. Specifically, we train these two networks with different amounts of paired training data and test their reconstruction accuracy on the same test dataset to evaluate their generalization abilities. The sizes of the training data sets are 50, 100, 300, 500, 1000, and 3000, and the test dataset size is 1000. Figure 3e shows a statistical analysis in terms of boxplots of obtained Chamfer distances. We observe that the performance of our method decreases slowly as the amount of training data is reduced; even with very few training data, for example, 50 samples, it still achieves an acceptable reconstruction accuracy; although, the stability of the imaging performance decreases. In contrast, the performance of the end‐to‐end reconstruction network drops exponentially with the reduction of training data. As the amount of training data increases, the end‐to‐end network's performance gradually aligns with our method. We hypothesize that the end‐to‐end reconstruction network is more prone to overfitting in situations of data scarcity. In contrast, the pretrained MGM can utilize learned features as “common sense” to compensate for the lack of training data, better handling tasks with limited data and effectively preventing overfitting. Some selected results are shown in Note S10, Supporting Information. Overall, our inversion framework exhibits strong generalization capabilities in the sense that it achieves high reconstruction accuracies with very little training data. This means that we can indeed build physical adapters at a low training cost, making the framework versatile and suitable for data‐limited application scenarios.

### 2.4 Experimental Results of 4D Microwave Meta‐Imaging

**Figure 4:**
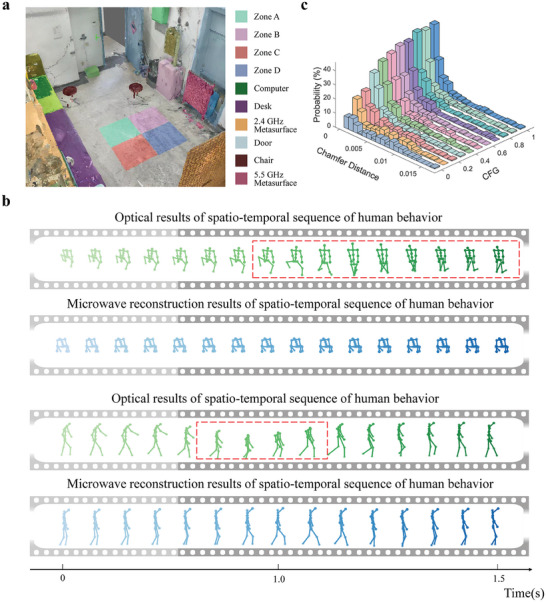
Selected experimental results for 4D microwave meta‐imaging. a) 3D visual–semantic map of the indoor environment. b) Two representative reconstructions of 1.5 s long 4D human skeletons obtained with stereo optical camera (first and third lines) and our 2.4 GHz microwave meta‐imaging system (second and bottom lines). Incoherent or meaningless frames in the optical results are marked with red dashed lines; they are well resolved with our microwave meta‐imaging strategy. c) Histograms of CD of microwave reconstructions with different values of CFG. Here, each histogram is computed over 800 test samples. The reconstruction accuracy of 4D microwave meta‐imaging increases as the CFG increases because the bigger the CFG is, the more the prior information is imposed.

Finally, we experimentally consider a more challenging and realistic meta‐imaging scenario aimed at monitoring temporal‐spatial human behavior in the complex indoor environment. Unlike the 3D meta‐imaging problem from the previous section, the 4D problem takes as input a sequence of microwave raw measurements and outputs a sequence of 3D images. In our experiments, the subject is represented by a 17‐point 3D skeleton, and the indoor environment is characterized with a visual‐semantic map; see **Figure** **4**a; Note S11, Supporting Information. Again, no suitable pretrained MGM is publicly available. Hence, we construct the MGM based on a multimodal diffusion model. We collect 3000 training samples using a stereo optical camera, and each sample is annotated with a text sentence or an optical image. The CFG technique is again utilized to control the prior's importance. Similar to the 3D case, the physical adapter is trained based on Equation (5) due to the lack of an explicit measurement model. We use 18 random metasurface configurations for each measurement and the frame rate can reach 20 frames per second. Further details about the MGM and the physical adapter can be found in Notes S12 and S13, Supporting Information, respectively.

An important general challenge for 4D imaging is that adjacent frames are not guaranteed to be logically plausible, especially when parts of the body are obscured. Our inversion strategy holds an important potential in addressing this difficulty because it is capable of addressing the information‐limited raw measurements thanks to the learned “common sense” in the pretrained MGM. To illustrate this important feature of our technique, two sets of experiments are conducted with the aforementioned 2.4 GHz system. Two representative experimental results are provided in Figure 4b, and more results are recorded in Videos S1 and S2, Supporting Information. Herein, the 4D microwave reconstruction results are reported in the second and bottom lines; while, for comparison, corresponding optical results are provided in the first and third lines. Note that the subject's legs are obscured in the first line, causing jitter in the leg key‐points captured by the optical camera, resulting in an illogical extension of the legs into the ground, as marked by the red dashed box. Such issues can be resolved by our microwave meta‐imaging strategy, mostly thanks to the common sense encoded into the pre‐trained MGM. Similarly, there is a sudden shortening in the subject's height in the sequence of optical 3D skeletons in the third line, as marked by the red dashed box. Thanks to the pretrained MGM, this phenomenon does not arise in the microwave meta‐imaging results, keeping the subject's height consistent, as shown in the bottom row.

We also quantify the importance of the semantic prior for the 4D meta‐imaging performance. Selected results are reported in Figure 4c; Note S14, Supporting Information. Figure 4c displays the histograms of 4D microwave reconstructions of the human skeleton for different values of CFG over 150 test samples, demonstrating quantitatively a reduction in the inversion error from 13 to 7 cm. Moreover, we can see that, as shown in Note S14, Supporting Information, the reconstruction results become more precise as the amount of prior information increases. As the initial frame priors of *p*_4_ and *p*_3_ are the same, when CFG is set at 0.5, the guiding strength of the prior is minimal, making the text prior in *p*_4_ ineffective and resulting in a reconstruction nearly identical to that of *p*_4_, where the target is merely standing up from the chair. However, when CFG is increased to 1.0, the text prior becomes effective, leading to a more precise reconstruction result, showing the target stretching. Now, it can be observed from these results that, consistent with previous experiments, the prior is capable of compensating for the inadequate and noisy measurements, thereby improving the reconstruction accuracy.

## 3 Discussion

To summarize, we presented a semantic‐EM inversion method capable of incorporating multimodal semantic priors in a flexible and frugal manner. At the core of our technique is a pretrained system‐agnostic multimodal generator modeling the unknown's distribution irrespective of the physical measurement system. The multimodal generator is fed the multimodal semantic prior via a multimodal foundation model and the EM measurement results via a lightweight physical adapter. The training of the latter is specific to the considered measurement system and can make use of an existing forward model of the measurement operator or be trained in a purely data‐driven manner. Ultimately, the EM inversion becomes a straightforward generation process controlled by the physical measurements and the multimodal semantic prior. Interestingly, we demonstrate that our framework can also be operated in a semantic‐guided step‐by‐step manner by updating the multimodal semantic prior in every step with natural language commands or reconstruction results from the previous step. In the present paper, we have to train the multimodal generator ourselves; however, whenever a large‐capacity MGM is already publicly available, it is, of course, preferable to use it directly or to fine‐tune it. Our experimental results in the context of reconstructing human postures and motion in complex indoor settings have direct technological relevance to smart homes, next generation wireless networks integrating sensing and communications, and more generally, to metamaterial agents interacting in natural language with humans.

## 4 Experimental Section

### Inverse‐Scattering Modeling

A 2D inverse‐scattering configuration under a TM‐polarized monochromatic illumination *E*_in_ was considered. As sketched in Figure 2a, a nonmagnetic scatterer characterized by a relative permittivity distribution ε_r_(r) was located within the DoI, and *N*_in_ transmitters and *N*_s_ receivers were uniformly distributed along a circle Γ outside the DoI. The object was successively illuminated by the *N*_in_ transmitters, and the resultant scattered fields *E*_s_ were sequentially captured by the *N*_s_ receivers for each illumination. The governing equations read:^[^ ^57^ ^]^

(6) $$E_{s}\;\left(r\right)=\;j\omega \varepsilon_{0}\int_{\mathrm{DoI}}g\left(r,r^{\prime }\right)\chi \left(r^{\prime }\right)E_{t}\left(r^{\prime }\right)dr^{\prime },\;r\in \Gamma \;$$

(7) $$E_{t}\;\left(r\right)=E_{\mathrm{in}}\;\left(r\right)+j\omega \varepsilon_{0}\int_{\mathrm{DoI}}g\left(r,r^{\prime }\right)\chi \left(r^{\prime }\right)E_{t}\left(r^{\prime }\right)dr^{\prime },\;r\in \mathrm{DoI}$$

where $g\;\left(r,r^{\prime }\right)=\;-\frac{\omega \mu_{0}}{4}H_{0}^{\left(2\right)}\left(k_{0}\left|r-r^{\prime }\right|\right)$ represents the 2D Green's function in free space, $H_{0}^{\left(2\right)}$ is the Hankel function of the second kind and zeroth order, χ(r′) =ε_*r*_ (r′) − 1, and ω is the angular frequency. Inverse scattering aims at reconstructing the distribution of χ from the measurements *E_s_* and corresponding illuminations *E*_in_. Further details are provided in Note S2, Supporting Information.

### Microwave Meta‐Imager

The microwave meta‐imager is a low‐cost versatile sensing device with software‐defined multifunctionalities. In the authors'experiments, it was composed of a large‐aperture programmable metasurface, an Ettus USRP X310, an emitter antenna, a trio of receiving antennas, and a host computer. The host computer was responsible for selecting control patterns for the metasurface, transmitting these patterns via the FPGA module to the metasurface and coordinating the synchronization of the USRP's transmission and reception channels. The host computer, USRP, and metasurface were interconnected via Ethernet using the transmission control protocol (TCP), with additional I/O series communication between the USRP and the metasurface. The programmable metasurface was a 2D array of electronically‐controllable meta‐atoms, where each meta‐atom was with a size of 54  ×  54 mm^2^ for the 2.4 GHz device and 24.4  ×  24.4 mm^2^ for the 5.5 GHz device and integrated with a PIN diode. Thereby, each meta‐atom could be configured to two distinct EM reflection states by controlling the bias voltage of the PIN diode. In particular, the meta‐atom would experience a phase shift of ≈180° in the vicinity of the operating frequency when the PIN was switched from ON to OFF through. The bias voltages for the PIN diodes were managed by a micro‐control unit based on an FPGA with a 50 MHz clock rate. More details are provided in Note S6, Supporting Information.

### Classifier‐Free Guidance (CFG) Technique

In general, the CFG technique was developed for trading‐off the contribution of multimodal prior and measurement. In the authors’ implementation, the semantic prompt was randomly replaced with an empty string during the training of the MGM, indicating the absence of a prior. After the training was completed, the importance of the semantic prior was adjusted through the formula ${\bar{\varepsilon }}_{t}=\left(1-w\right)\;\varepsilon_{\theta }\left(z_{t},t\right)+w\varepsilon_{\theta }\left(z_{t},t,\alpha \right),$ where ${\bar{\varepsilon }}_{t}$ represents the final noise predicted by the diffusion model at *t*‐th step, *w* is the guidance coefficient, ε_θ_(*z*_t_,*t*) is the noise predicted by the diffusion model without condition at *t*‐th step, and ε_θ_(*z_t_*,*t*, α) is the noise predicted by the diffusion model with multimodal semantic embedding α at the *t*‐th step. More details are provided in Note S7, Supporting Information.

### Statistical Analysis

All experiments were performed in three or more replicates. Results were expressed as the mean ± standard deviation. For intergroup comparisons, repeated data were examined based on variance analysis. All numerical simulations of the programmable metasurface were performed using a commercial full‐wave EM simulator, CST Microwave Transient Simulation Package 2017. The network design, training, and computational tasks were all executed using the PyTorch library.

## Conflict of Interest

The authors declare no conflict of interest.

## Author Contributions

Y.C., H.Z., and J.M. contributed equally to this work. T.J.C., P.d.H., and L.L. conceived the idea and wrote the manuscript. Y.C., H.Z., and J.M. designed and developed the system and conducted the experiments. All authors participated in the data analysis and interpretation and read the manuscript.

## Code Availability

Code that supports the findings of this study is available upon reasonable request from L.L.

## Supporting information



Supporting Information

Supplemental Video 1

Supplemental Video 2

## Data Availability

The data that support the findings of this study are available from the corresponding author upon reasonable request.
