# Supplementary material for: Semantic–Electromagnetic Inversion With Pretrained Multimodal Generative Model
Source: Adv Sci (Weinh). 2024 Sep 9;11(42):2406793. doi: 10.1002/advs.202406793 (PMC11558082; doi:10.1002/advs.202406793)
Supplement: Supplementary file 1 — Supporting Information [file ADVS-11-2406793-s002.docx]

**Supplementary Information for**

**Semantic-Electromagnetic Inversion with Pretrained Multimodal Generative Model**

Yanjin Chen^1+^, Hongrui Zhang^1+^, Jie Ma^1+^, Tie Jun Cui^2,3^, Philipp del Hougne^4^, and Lianlin Li^1,3^

^1^ State Key Laboratory of Advanced Optical Communication Systems and Networks,

School of Electronics, Peking University, Beijing 100871, China

^2^ State Key Laboratory of Millimeter Waves, Southeast University, Nanjing 210096, China

^3^ Pazhou Laboratory (Huangpu), Guangzhou, Guangdong 510555, China

^4^ Univ Rennes, CNRS, IETR - UMR 6164, F-35000 Rennes, France

^+^ Equal contributions.

**Outline:**

**Supplementary Notes S1.** Supplementary Explanation of the Overall Framework and Components

**Supplementary Notes S2.** Setups of 2D EM Inverse Scattering Experiments

**Supplementary Notes S3.** Details of MGM and Training Algorithm in the 2D EM inverse problem

**Supplementary Notes S4.** Details of Physical Adapter and Flowchart of the Physics-driven Scheme in the 2D EM inverse problem

**Supplementary Notes S5.** Comparison with Deep Image Prior-based methods

**Supplementary Notes S6.** System Configuration of the Microwave Metasurface Camera and Details of 3D Compressive Microwave Meta-imaging Experiment

**Supplementary Notes S7.** Details of MGM and Training Algorithm in the 3D Microwave Meta-imaging Experiment

**Supplementary Notes S8.** Details of Physical Adapter in the 3D Microwave Meta-imaging Experiment

**Supplementary Notes S9.** Supplementary Results on 3D Microwave Meta-imaging for 5.5 GHz Systems

**Supplementary Notes S10.** Supplementary Results with Comparison Experiment with End-to-end Network

**Supplementary Notes S11.** Details of 4D Compressive Microwave Meta-imaging Experiment

**Supplementary Notes S12.** Details of MGM and Training Algorithm in the 4D Microwave Meta-imaging Experiment

**Supplementary Notes S13.** Details of Physical Adapter in the 4D Microwave Meta-imaging Experiment

**Supplementary Notes S14.** Supplementary Results on the Effects of the Importance of Semantic Prior on Performance in 4D Meta-imaging

**Supplementary Notes S1. Supplementary Explanation of the Overall Framework and Components**

**1. Overall Framework.**

We propose a method for solving the semantic-electromagnetic (EM) inversion problem that flexibly combines multimodal semantic priors. The overall framework consists of the following main components: a Multimodal Generative Model (MGM), a Multimodal Foundation Model (MFM), and a Physical Adapter. The core of our technique is a pretrained, system-agnostic Multimodal Generative Model (MGM) that can model the "natural" distribution of the target without relying on specific physical measurement systems. The Multimodal Foundation Model (MFM) is also pretrained and is responsible for encoding multimodal data into multimodal semantic prior embeddings $\alpha$, which are then input into the Multimodal Generative Model as prior information.

The training of the Physical Adapter is specific to the measurement system under consideration and can be carried out using existing forward models or entirely data-driven methods. When the forward model of the measurement system is known, we can optimize the parameters of the Physical Adapter according to Equation (4) and obtain the unknown variable x, a strategy similar to Deep Image Prior (DIP). In this case, we do not need training data, and each test data requires an online solution of the corresponding Physical Adapter parameters. When the forward model of the measurement system is unknown, we can train the parameters of the Physical Adapter online using the collected paired training data according to Equation (5). Once trained, the Physical Adapter can be used offline for testing.

**2. Components.**

The overall framework consists of the following main components: the Multimodal Generative Model (MGM), the Multimodal Foundation Model (MFM), and the Physical Adapter.

**(1) Multimodal Generative Model (MGM).**

The MGM is the core component of our framework. It captures the "natural" distribution of the target object, that is, the "common sense" about the target, and outputs reconstruction results through the generation process. During the pretraining phase, the MGM is trained using matched unknowns and corresponding text and image priors without the need for corresponding EM measurement data. Once trained, the MGM is "frozen", meaning its weights are no longer updated, and it can be used with different Physical Adapters.

**Input:** During pretraining, the inputs are the multimodal semantic prior embeddings $\alpha$ from the MFM and a random variable $z$. When combined with the Physical Adapter, an additional input from the Physical Adapter, the low-level information embedding $\Delta\alpha$, is added.

**Output:** The MGM's output is the result $x$. During pretraining, this result is derived from the input multimodal prompts. When combined with the Physical Adapter, the result is further controlled by the measurement data.

**(2) Multimodal Foundation Model (MFM).**

The MFM encodes multimodal prior data into multimodal semantic prior embeddings $\alpha$. These priors can be a combination of text, images, or other forms. The MFM's role is to provide prior information to the MGM, enabling the generative model to produce more accurate reconstruction results based on these priors.

**Input:** Various modal prior $p$.

**Output:** Multimodal semantic prior embeddings $\alpha$.

**(3) Physical Adapter.**

The Physical Adapter is responsible for embedding the EM measurement data into low-level information embeddings $\Delta\alpha$ so that it can control the Multimodal Generative Model to generate targets consistent with the EM measurement data. Its training can be based on known physical forward models (if available) or on matched training examples in a supervised learning manner when no forward model is available. The design of the Physical Adapter allows our framework to adapt to different physical measurement systems with minimal training data. Its combination with the MGM is based on ControlNet.

**Input:** EM measurement $y$.

**Output:** Low-level information embeddings $\Delta\alpha$.

**3. Training Process.**

The training process of this framework is divided into two stages.

**(1) Pretraining Phase.**

In the pretraining phase, we need to determine the imaging target and the types of multimodal priors and then establish the Multimodal Generative Model and the Multimodal Foundation Model based on this information. If there are existing generative models and multimodal foundation models (such as the CLIP model), we can directly use these models. If not, we need to collect matched targets and corresponding multimodal prior training data to train the Multimodal Generative Model and the Multimodal Foundation Model.

**(2) Formal Training Phase.**

In the formal training phase, we need to train the Physical Adapter. If the forward model of the measurement system is known, we can adopt a strategy similar to DIP, which does not require collecting training data. Instead, we directly optimize the parameters of the Physical Adapter online using the measurement data to obtain the corresponding imaging result. In this case, the optimized Physical Adapter parameters are specific to this measurement data, and new measurement data requires re-optimization to obtain new Physical Adapter parameters. If the forward model of the measurement system is unknown, we can use a data-driven approach to train the Physical Adapter. We can collect some paired measurement data and targets under the measurement system as training data to train the Physical Adapter online. Once trained, the Physical Adapter can be used for all measurement data under that measurement system without retraining.

**4. Advantages of the Framework.**

(1) Flexibility of Multimodal Priors: Our framework can handle various forms of prior information (text, images, etc.) and flexibly combine these priors to improve reconstruction accuracy.

(2) Adaptation to Different Physical Measurement Systems: By training different Physical Adapters, our framework can adapt to various physical measurement systems without retraining the entire generative model.

(3) Reduced Training Data Requirements: Since the MGM has already learned the "common sense" distribution of the target object during pretraining, training the Physical Adapter requires only a small amount of paired data.

(4) Generation of Solution Distributions: Our framework views inversion as a generative task and can generate a distribution of solutions rather than a single solution. By sampling from this distribution using the random variable z, multiple possible solutions can be obtained, better capturing the potential features of the target object.

**Supplementary Notes S2. Setups of 2D EM Inverse Scattering Experiments**

We use the numbers in the MNIST^[1]^ dataset to model 2D scatterers as the object in the EM inverse scattering problem. The size of the domain of interest (DoI) is 1.28m×1.28m, which is discretized into 64×64 pixels with the size of 0.02m×0.02m. The operating frequencies are 100MHz, 300MHz and 500MHz. A total of four transmitters and eight receivers are uniformly placed on a circle with a radius of 2m, as shown in **Supplementary Figure S2.1**. Thus, the received microwave measurement contains 32 complex values. In this case, the scatterers are assumed to be isotropic and lossless, and the background medium is set as air. The relative permittivity of the scatterer is randomly chosen in the range of [1.5, 2.0]. We used CG-FFT^[2]^ as the forward solver to calculate the scattered field data.

**
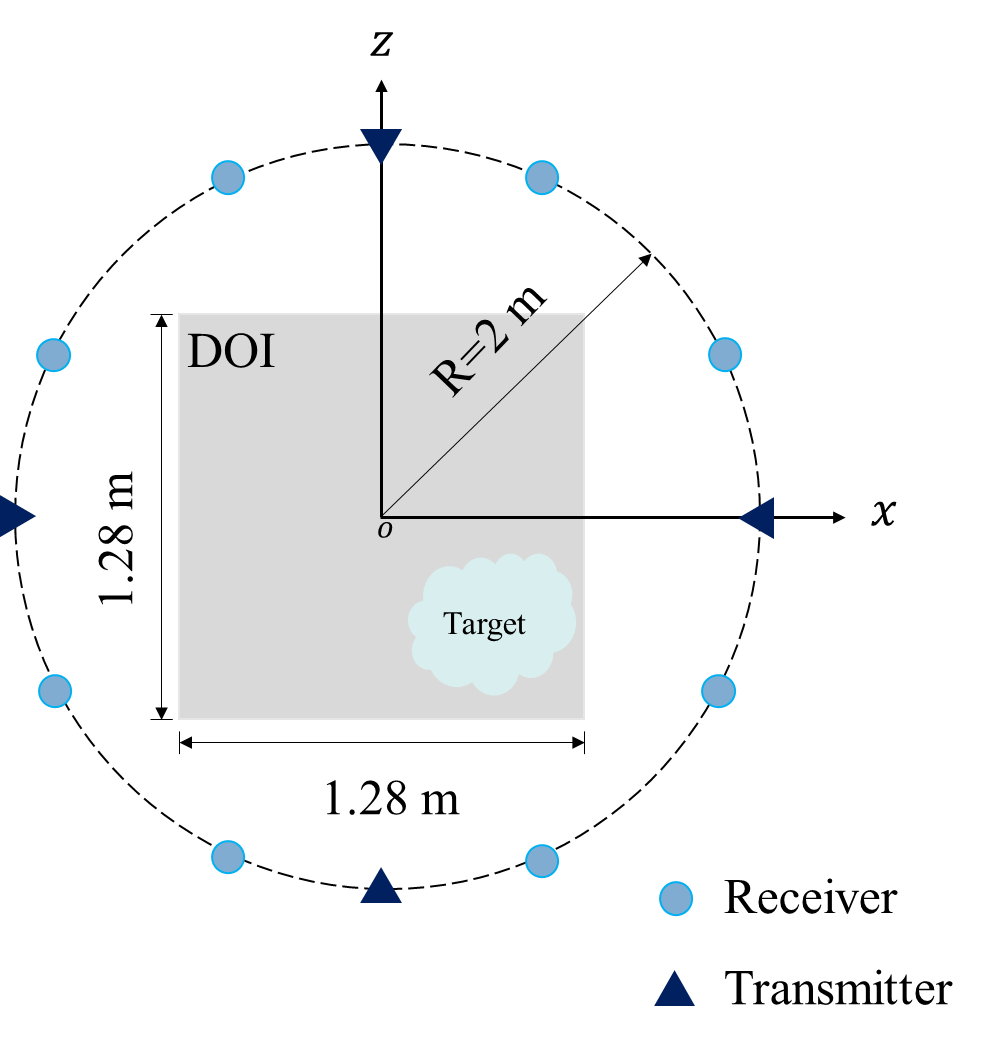
**

**Supplementary Figure S2.1** | **Model configuration for the 2D EM inverse problem scenario.**

**Supplementary Notes S3. Details of MGM and Training Algorithm in the 2D EM inverse problem**


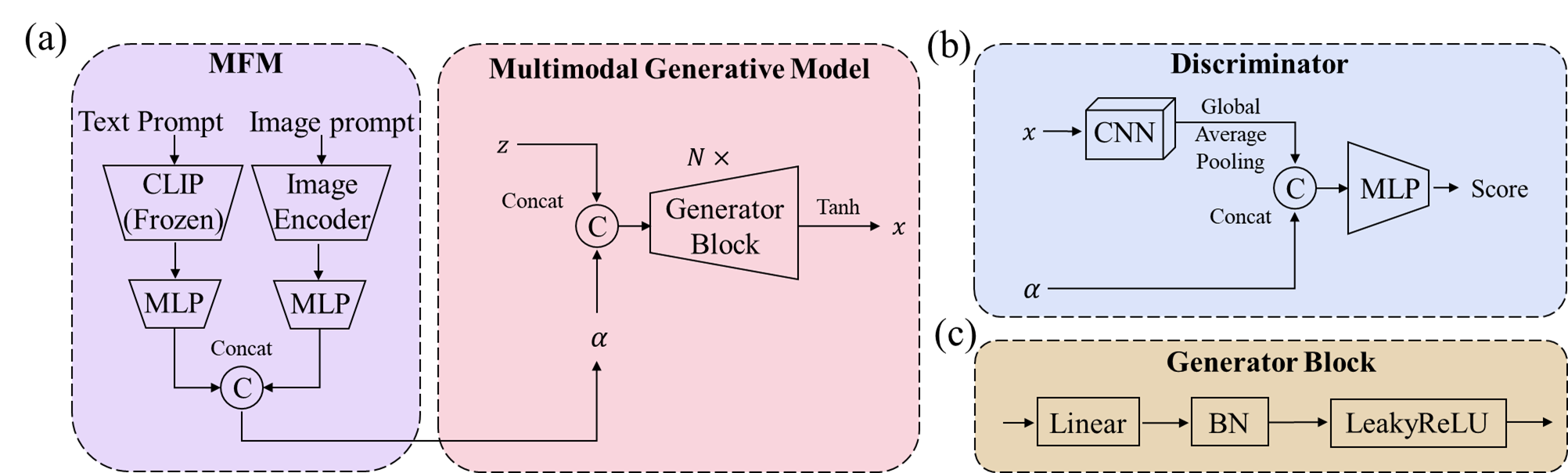


**Supplementary Figure S3.1** | **Internal composition of the MGM for 2D EM inverse scattering problem.** **(a)** The architecture of the ‘MGM’ and the ‘Multimodal Foundation Model’. **(b)** The architecture of the ‘Discriminator’. **(c)** The architecture of the ‘Generator Block’. Here, MFM stands for the multimodal foundation model, MGM stands for the multimodal generative model, CLIP is the text encoder in the Contrastive Language-Image Pre-training model, MLP is the multilayer perceptron which is composed by cascaded fully connected neurons and activation functions (LeakyReLU), CNN represents a convolutional neural network in two dimensions, BN represents the batch normalization layer.

In our study on the 2D EM inverse scattering problem, the multimodal generative model (MGM) is trained using the framework of Generative Adversarial Network (GAN)^[3]^, which is shown in **Supplementary Figure S3.1(a)**. The MGM is composed of the multiple generator blocks. Its inputs are a latent variable $z$ and a multimodal semantic embedding $\alpha$, which are concatenated together to generate the normalized target image $x$ via multiple generator blocks and Tanh activation function. The latent variable $z$ is a random variable sampled from the standard Gaussian distribution. The multimodal semantic embedding $\alpha$ comes from the multimodal foundation model (MFM), which integrates information from multimodal prompts (text and images in this experiment). The multimodal foundation model consists of a frozen pre-trained CLIP model^[4]^, a image encoder and the multi-layer perceptron (MLP)^[5]^. It utilizes the text encoder in the CLIP model and image encoder to transform the text prompt and image prompt into text embedding and image embedding, respectively. These two embeddings go through two MLPs respectively and then concatenated together to form the multimodal semantic embedding $\alpha$. The CLIP model, or Contrastive Language-Image Pre-training model, was developed by the OpenAI team to investigate which factors enhance robustness in computer vision tasks and to test the model’s zero-shot generalization ability across various image classification tasks. This model later became foundational for many text-to-image generation models, such as the well-known Stable Diffusion^[6]^. CLIP was trained on a wide range of publicly available image caption datasets, including data scraped from a few websites and common image datasets like YFCC100M^[7]^. **Supplementary Figure S3.1(b)** presents the discriminator of the GAN, consisting of the convolutional neural network (CNN) ^[8]^, and MLP. Its inputs include a normalized target image $x$ and a multimodal semantic embedding $\alpha$. The normalized target image $x$ is processed through the CNN and converted into the target embedding via global average pooling. The target embedding and the multimodal semantic embedding $\alpha$ are concatenated to form the joint embedding, which is then processed by an MLP to produce a score that reflects the degree of match between the normalized target image $x$ and the multimodal prompt. **Supplementary Figure S3.1(c)** shows the structure of the generator block, which consists of a linear layer, a batch normalization layer, and a LeakyReLU^[9]^ activation function.

This GAN training method uses the WGAN-GP^[10]^ approach. Here is the loss function used during training:

$L_{D}=E_{x,\alpha,z}[[-D\left( x,\alpha\right)+D\left( \mathcal{G}\left( \alpha,z \right),\alpha\right)]+\lambda\left[ \left\| \nabla_{\mathcal{G}\left( z,\alpha\right)}D\left( \mathcal{G}\left( \alpha,z \right),\alpha\right) \right\|_{2}-1 \right]^{2}].$ (1a)

$L_{\mathcal{G}}=E_{x,\alpha,z}[-D\left( \mathcal{G}(\alpha,z),\alpha\right)].$ (1b)

Here, $\alpha=MFM(p)$ is the multimodal semantic embedding, $p$ is the multimodal prompt, $D$ represents the discriminator, $\mathcal{G}$ represents the MGM and $x$ is the normalized target image. The latent variable $z$ follows a standard normal distribution, and $\lambda$ is the weight for the gradient penalty. The discriminator's loss function (1a) aims to increase the score for real samples and decrease the score for generated samples, with an added gradient penalty; the MGM is frozen during the training of the discriminator. The MGM's loss function (1b) aims to increase the score for generated samples to confuse the discriminator; the discriminator is frozen during the training of the MGM.

In the experiment of the 2D EM inverse scattering problem, all real target images are normalized to the range [-1, 1] before training. The outline in the image prompts are obtained via Holistically-Nested Edge Detection (HED)^[11]^, and sketch prompts through a combination of manual and automatic annotation. Sparse, smooth, and sharp edge image prompts are produced through specific algorithm. When there is no text prompt, the text is set to the “unknown” string; when there is no image prompt, the image is set to an all-zero image. The CLIP model, used as a pre-trained model, remains frozen throughout the training, outputting original text embeddings of dimension 768. In the MFM, both text and image embeddings are reduced to a dimension of 128. And in the MGM, the dimension of the latent variable $z$ is 128, and the final target image output size is 64×64 pixels. The MGM contains 4 generator blocks. In the discriminator, the target image embedding is set to 512, and the final output is a scalar score assessing the image's authenticity. Both the discriminator and the generator use the LeakyReLU activation function with a hyperparameter of 0.2. The training ratio between the discriminator and the generator is set at 5:1. The total number of training data is 50000. The batch size is 128, the learning rate is 0.0002, and the optimizer is Adam with $\beta_{1}=0.9$ and $\beta_{2}=0.99$. A total of 3000 training epochs are conducted.

**Supplementary Notes S4. Details of Physical Adapter and Flowchart of the Physics-driven Scheme in the 2D EM inverse problem**


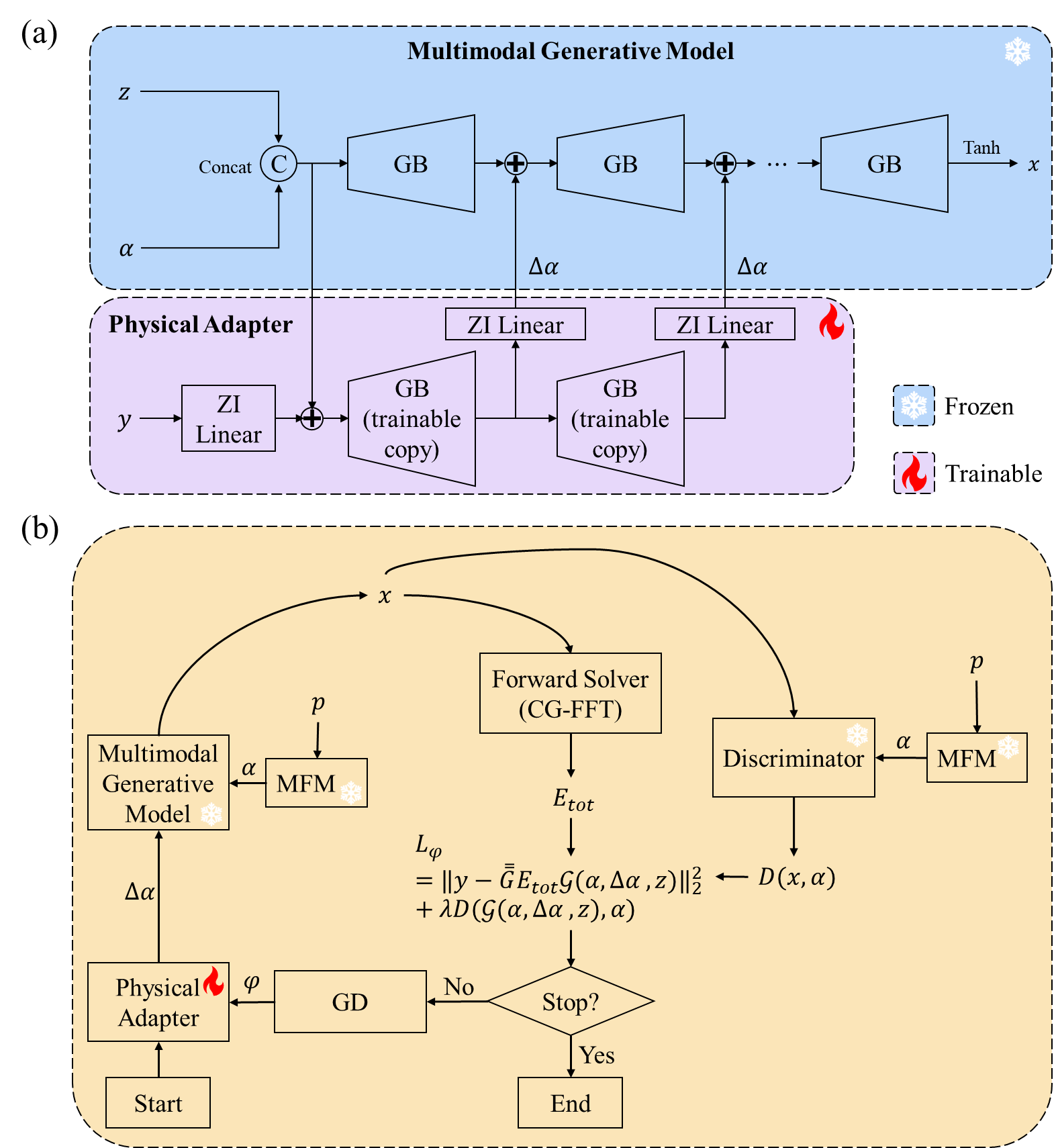


**Supplementary Figure S4.1** | **The physical adapter and the flowchart of the physics-driven scheme used in the 2D EM inverse problem.** **(a)** The physical adapter in the 2D EM inverse scattering experiment. **(b)** The flowchart of the physics-driven scheme in the 2D EM inverse scattering experiment. Here, ‘GB’ represents the generator block, as shown in **Supplementary Figure S3.1(c)**; ‘ZI’ means zero initialization; ‘trainable copy’ means that the parameters initialized at the beginning of training are those of the original module; ‘MFM’ is the multimodal foundation model; ‘GD' represents the gradient descent method, ‘CG-FFT’ represents the conjugate-gradient fast Fourier transform method.

We employ ControlNet^[12]^ from stable diffusion as the physical adapter, which manipulates the input conditions of neural network blocks to further control the overall behavior of the network. So, the physical adapter utilizes a slim network consisting of several "trainable copies" of the generator block (GB) and "zero-initialized" linear layers, as shown in **Supplementary Figure S4.1(a)**. The input, physical measurements $y$, are dimensionally matched through a "zero-initialized" linear layer before being combined with the input of the MGM, which is obtained by concatenating the hidden variable $z$ and the multimodal semantic embedding $\alpha$. It then passes through multiple "trainable copies" and "zero-initialized" linear layers, ultimately inputting the residual low-level parameter $\Delta\alpha$ into the backbone network, i.e., the MGM. "Trainable copies" are replicas of the original network blocks with identical, trainable parameters. "Zero-initialized" layers start with weights and biases initialized at zero, which are gradually optimized during training.

**Supplementary Figure S4.1(b)** shows the flowchart of the physics-driven scheme used in the 2D EM inverse scattering experiment, which is an iterative loop framework. First, the parameters $\varphi$ of physical adapter are initialized, and a solution $x$ is generated through the MGM, whose inputs are the residual low-level parameter $\Delta\alpha$ output by the physical adapter and the multimodal semantic embedding $\alpha$ output by the MFM. Then, through the forward solver, here referred to the conjugate-gradient fast Fourier transform method (CG-FFT) method, the total field $E_{tot}$ at the DoI is obtained, and a matching score $D(x,MFM(p))$ between the solution $x$ and multimodal prompt $p$ is obtained through the discriminator. These outputs together form the objective function:

$L_{\varphi}={\|y-GE_{tot}\mathcal{G}(MFM(p),\mathcal{M}_{\varphi}(y),z)\|}_{2}^{2}+\lambda D(\mathcal{G}(MFM(p),\mathcal{M}_{\varphi}(y),z),MFM(p)).$ (2)

where $\lambda$ is a weight parameter used to adjust the importance of prior information; $G$ represents the Green's function, and $GE_{tot}$ is the measurement model $\mathcal{L}$. Next, it is determined whether the stopping condition has been met. If so, the loop exits; if not, the objective function is optimized by gradient descent (GD) to obtain new parameters $\varphi$, proceeding to the next iteration step and repeating this process. In the experiment, GD method is implemented using Pytorch's^[13]^ Adam^[14]^ optimizer, and $\lambda$ is set at 1e^-10^.

**Supplementary Notes S5. Comparison with Deep Image Prior-based methods**

**
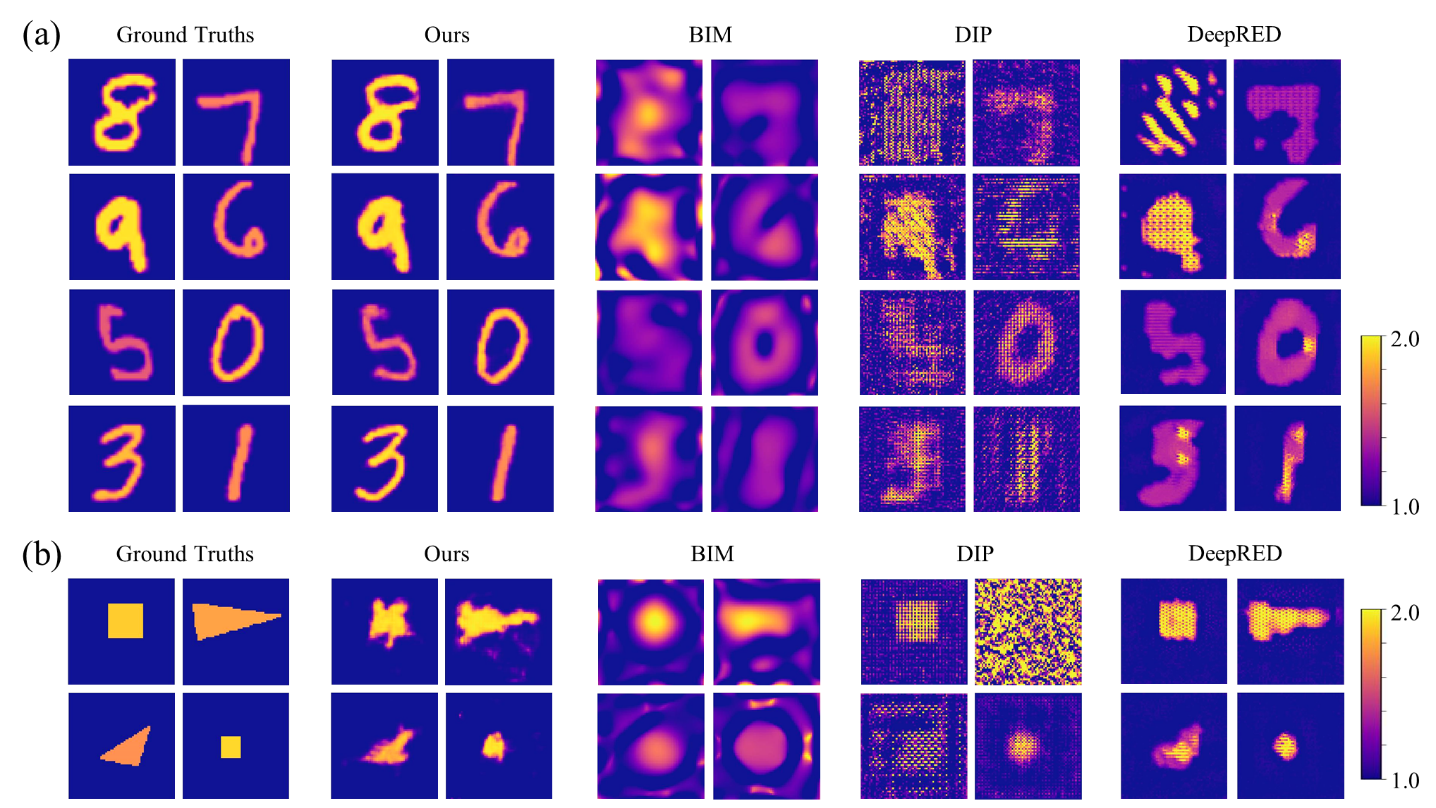
**

**Supplementary Figure S5.1** | **Comparison of our framework, BIM, DIP and DIP-RED. (a)** Comparison on digit-like samples. **(b)** Comparison on geometric samples.

Deep Image Prior (DIP)^[15]^ is an untrained network prior method used to solve image inverse problems, primarily used in image denoising and repair tasks. This method does not depend on large training data and can work directly with a damaged image. Its primary goal is to minimize the difference between the network output image and the damaged image. DIP operates on the principle of spectral bias, meaning neural networks more readily adapt to low-frequency information than high-frequency information during the fitting process. This implies that the network first captures the global structure and main features of the image and gradually addresses more high-frequency details (such as noise and texture). By stopping the iteration process at the appropriate time, a balance can be found between reconstruction quality and overfitting. Moreover, DIP considers neural networks as a form of implicit regularization, with different network architectures offering different effects of regularization. For example, the spatial translation invariance and layer-by-layer abstraction of deep convolutional neural networks allow them to learn the prior information of natural images from a single damaged image. With the development of DIP, the DeepRED^[16]^ method was introduced, which adds regularization by denoising (RED) to the original DIP, further enhancing the method’s effectiveness. The DIP method has also been extended to other inverse problem areas, such as optical computational imaging^[17]^. Of course, we can also set the network parameter directly as the unknown $x$, thereby transforming this method into the traditional Born iterative method (BIM) ^[18]^. Given the similarities between DIP and our physics-driven framework, we compare our framework with BIM, DIP and DeepRED in the 2D EM inverse scattering experiment.

In BIM, we solve for the unknown $x$ by optimizing the following cost function:

$L_{x}=\left\| y-\mathcal{L}(x;E_{in}) \right\|_{2}^{2}.$ (3)

where $\mathcal{L}$ is the measurement model, $E_{in}$ is the incident field, and $y$ is the measured scattered field.

In DIP, we obtain the network parameters by optimizing the following cost function:

$L_{\theta}=\left\| y-\mathcal{L}(T_{\theta}(z);E_{in}) \right\|_{2}^{2} s.t. x=T_{\theta}\left( z \right).$ (4)

where $T_{\theta}$ represents a neural network with parameters $\theta$, specifically, a U-Net, and $z$ is a random variable that remains unchanged throughout the process and matches the dimensions of the unknown $x$.

In DeepRED, we obtain the network parameters by optimizing the following cost function:

$L_{\theta}=\left\| y-\mathcal{L}(T_{\theta}(z);E_{in}) \right\|_{2}^{2}+\lambda{[T_{\theta}(z)]}^{T}\left[ T_{\theta}\left( z \right)-R_{\varphi}\left( T_{\theta}\left( z \right) \right) \right] s.t. x=T_{\theta}\left( z \right).$ (5)

where $R_{\varphi}$ is a denoiser with pre-trained parameters $\varphi$. We use U-Net as the denoiser, training it on 10,000 samples from the MNIST dataset with additive white Gaussian noise (AWGN) randomly introduced at SNRs from 30dB to 5dB. $\lambda$ is the regularization parameter. $T_{\theta}$ is consistent with that used in DIP.

As shown in **Supplementary Figure S5.1 (a)**, it is evident that our framework achieves the highest similarity to the actual target in terms of reconstruction results. The BIM method, while able to adequately outline the basic contours of the target, often overestimates the target's volume and underestimates its relative permittivity. The results from the DIP method are characterized by grid-like spots, primarily due to the characteristics of the convolutional kernels, indicating that the prior information embedded within the structure of convolutional neural networks alone is insufficient to address the ill-posed nature of inverse problems. The DeepRED method, by incorporating denoising regularization, successfully resolves the noise issues found in DIP, resulting in a smoother reconstructed target. However, despite the inclusion of RED, DeepRED still captures only the general shape of the target, demonstrating its limitations in addressing the ill-posedness of the problem. Combining these results, we can conclude that among these four methods, only the MGM used in our framework effectively alleviates the ill-posed nature of inverse problems, thereby enabling more accurate target reconstruction. Given the generalization issues of the MGM, we compare the four methods on geometrical samples. As shown in **Supplementary Figure S5.1 (b)**, our method not only performs better than BIM and DIP but is also competitive with DeepRED.

**Supplementary Notes S6. System Configuration of the Microwave Metasurface Camera and Details of 3D Compressive Microwave Meta-imaging Experiment**

**Introduction of the system for** **compressive microwave imaging.**


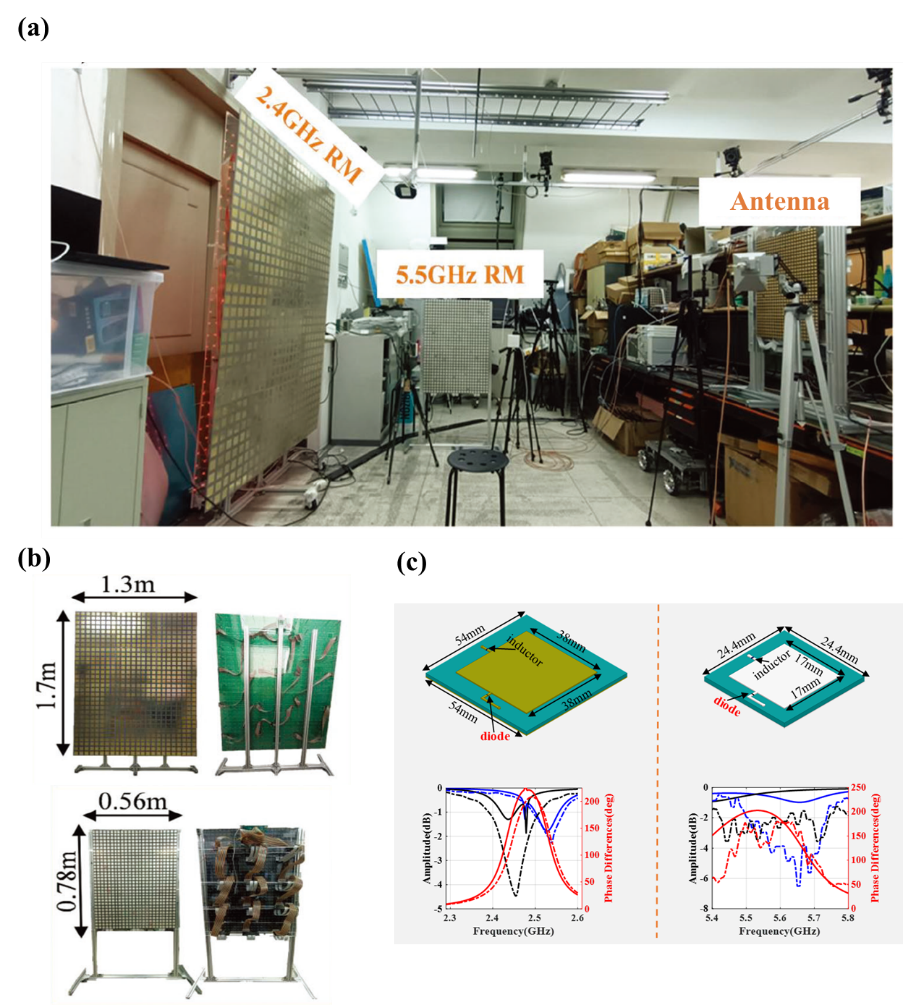


**Supplementary Figure S6.1** | **System Configuration of the Microwave Metasurface Camera. (a)** The microwave metasurface camera laboratory setup. **(b)** Physical metasurfaces at 2.4GHz and 5.5 GHz. **(c)** The sketched map and experimental and simulated results of magnitude-frequency and phase-frequency responses of the designed meta-atom.

The electromagnetic sensing system based on intelligent metamaterials used in this experiment covers two different frequency bands of intelligent metamaterials, which are 2.4GHz and 5.5GHz, respectively. The system consists of two parts: the data acquisition hardware centered around intelligent metamaterials and the data post-processing software centered around algorithms. At the hardware level, the system consists of a host and two subsystems centered around programmable metamaterials, which operate at around 2.4GHz and 5.5GHz, respectively. The host computer serves as the brain of the entire system, mainly responsible for real-time data processing, online decision-making, computing metamaterial control codes, and communicating with other hardware via Ethernet. Each subsystem centered around metamaterials consists of a metamaterial, a field programmable gate array (FPGA), a commercial software-defined radio device (Ettus Universal Software Radio Peripheral (USRP) X310), and a pair of horn antennas. The USRP and horn antennas are responsible for generating probing signals and capturing target echoes. In this work, for both subsystems, we chose chirp waveform as the probing signals, i.e.,$s\left( t \right)=exp(j\left( 2\pi f_{c}t+\pi Kt^{2} \right))$, $0\leq t\leq T$, where $j=\sqrt{-1}$, $f_{c}=2.424$GHz is the carrier frequency, $K=B/T$ denotes the sweep rate of the chirp, B=50MHz is the frequency bandwidth, and T=10μs is the Chirp pulse duration. At the software level, the algorithms installed on the host computer are roughly divided into three groups. One group is used to control the software-defined hardware, such as USRP and FPGA, the second is for signal-level preprocessing and feature extraction, while the third group is for machine learning-driven data post-processing. The experimental setup in our laboratory is depicted in **Supplementary Figure S6.1(a)**.

The array structures of the two types of intelligent metasurfaces are illustrated in **Supplementary Figure S6.1(b)**. Each metasurface is precisely controlled through a Micro-Control Unit (MCU) based on FPGA and maintains efficient communication with the host computer. The entire metasurface array is composed of 3×4 subpanels, with each subpanel consisting of 8×8 identical units. The specific scale of the entire array is 24×32, corresponding to array aperture sizes of $1.7\times1.3m^{2}$ and $0.781\times0.586m^{2}$, respectively. When the intelligent metasurfaces are in operation, the host computer sends precise instructions to all subpanels via FPGA, enabling simultaneous control of 768 PIN diodes. All subpanels of the intelligent metasurfaces share the same clock signal, with a speed of up to 50 MHz, and the ideal switching time of PIN diodes can reach 2.5 microseconds.

Each unit structure of the metasurface mainly includes rectangular metal resonant patches, phase-shifting lines, dielectric substrates, and metal reflectors. A PIN diode is integrated between the metal patch and the phase-shifting line of each unit, and the phase-shifting line and the metal ground plane are connected through metal vias. By electronically controlling the "on" and "off" states of the PIN diode, the metasurface can achieve 180° phase reversal of reflected electromagnetic waves.

For the 2.4GHz metasurface unit, there are two dielectric substrate layers: the top dielectric layer is F4B with a relative dielectric constant of 2.55 and a loss tangent of 0.0019, and the bottom dielectric layer is FR4. The PIN diode used is the SMP1345-079LF model, and an RF choke with an inductance of L = 33nH is used to suppress AC coupling between the diode and the ground plane. The structure of the 5.5GHz metasurface unit is similar to that of the 2.4GHz unit, but its dimensions have been correspondingly reduced to accommodate the higher frequency. We have comprehensively verified the electromagnetic performance of the electronically controllable metasurface units through simulation and experimental means. Firstly, precise simulation designs of the metasurface units were conducted using commercial electromagnetic simulation software CST 2017 to ensure the theoretical feasibility of the unit models. Subsequently, the metasurface units were processed and tested based on the simulation models. For the 2.4GHz metasurface unit, detailed testing and analysis of its S-parameters were performed using standard waveguides. Due to the stability of the design structure of the 5.5GHz metasurface unit, the performance testing of the processed metasurface array was directly conducted using the free-space array measurement method. Specifically, the reflection phase of the metasurface unit undergoes a 180° flip when the PIN diode switches from "on" to "off" or from "off" to "on" within the operating frequency range. This key feature can be controlled directly by adjusting the external voltage on the PIN diode from 12V to 0V, thereby achieving electronic control of the electromagnetic performance of the metasurface. The unit design models and their electromagnetic response characteristics for both types of intelligent metasurfaces are shown in **Supplementary Figure S6.1(c)**.

**Dataset collection and preparation.**

The microwave imaging experiment is deployed in a real indoor environment, leading to significant noise in the microwave measurement data, especially around 2.4GHz. Therefore, to obtain satisfactory imaging results, we apply denoising and temporal averaging filtering to the raw microwave signals received by the receiving antenna, resulting in microwave data with dimensions of 20×32 at the 2.4GHz frequency band. Here, "20" represents the number of randomly generated control codes in our experiment, and "32" represents the most significant 32 feature points under each code mode, including the real and imaginary parts. In contrast, at the 5.5 GHz frequency band, our microwave data dimension is 20×64.

In the compressing microwave imaging experiments under 2.4GHz and 5.5 GHz system, we choose the form of 3D point cloud to describe the target. To obtain optical data matching the microwave data for supervision, we integrate a commercial binocular optical camera named ZED2 into the system for synchronized data collection with microwaves. Specifically, the ZED2 camera can capture high-definition indoor photos and three-dimensional skeleton information in real-time. Subsequently, we process this information using the open-source SMPL-X^[19]^ model to obtain three-dimensional human body meshes, and finally, we sample them to obtain the required amount of point cloud data. The point cloud data used in the experiment contains 2048 points.

**Supplementary Notes S7. Details of MGM and Training Algorithm in the 3D Microwave Meta-imaging Experiment**


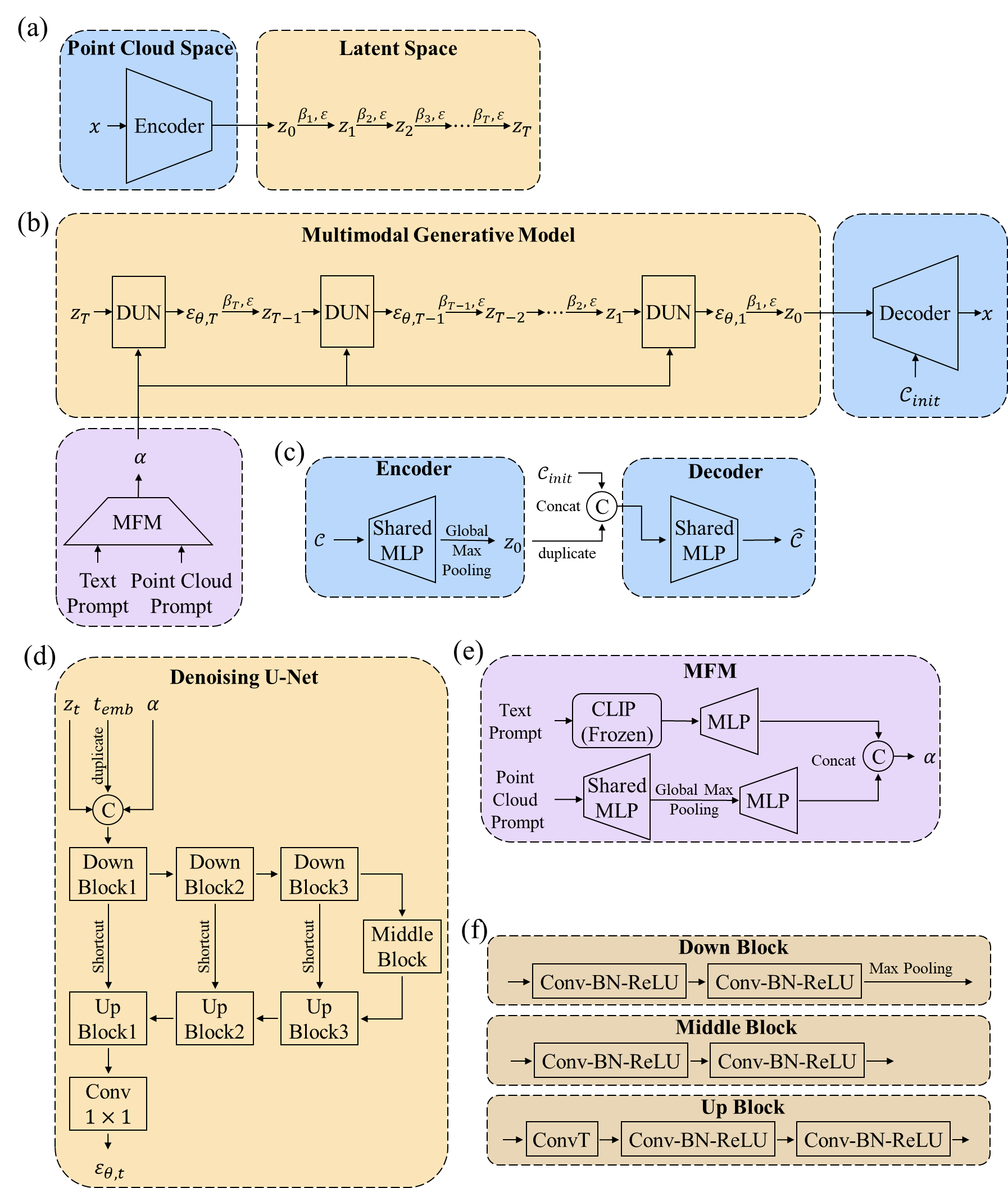


**Supplementary Figure S7.1** | **Internal composition of the MGM for 3D microwave meta-imaging experiment.** **(a)** Diffusion processes of the diffusion model. **(b)** Overall structure of the MGM and the reverse diffusion process of the diffusion model. **(c)** The architecture of the ‘Autoencoder’. **(d)** The architecture of the ‘Denoising U-Net’. **(e)** The architecture of the ‘Multimodal Foundation Model’. **(f)** The architecture of the ‘Down Block’, ‘Middle Block’ and ‘Up Block’. Here, ‘DUN’ represents the Denoising U-Net; ‘MFM’ represents the Multimodal Foundation Model; Shared MLP represents an MLP with shared weights for each point; Conv$1\times1$ represents a two-dimensional convolution layer with a kernel size of 1; Conv-BN-ReLU denotes the module consisting of a 2D convolutional layer, a batch normalization layer and a ReLU activation function; ConvT represents a transposed convolution layer that doubles the size of the feature map.

In the 3D microwave meta-imaging experiment, we employ a diffusion model^[20]^ as the MGM, with the specific network structure displayed in **Supplementary Figure S7.1**. **Supplementary Figure S7.1(a)** illustrates the diffusion process of the diffusion model. Initially, the point cloud $x$ is encoded into the latent variable $z_{0}$ by an encoder. Then, the diffusion process occurs in the latent space, where noise is incrementally added to the latent variable $z_{0}$ until the $T$-th step. At step $t$, the formula for adding noise is as follows:

$z_{t}=\sqrt{1-\beta_{t}}z_{t-1}+\sqrt{\beta_{t}}\varepsilon.$ (6)

where $\beta_{t}$ controls the variance of the Gaussian noise at step $t$, and $\varepsilon$ is a random variable sampled from the standard Gaussian distribution. **Supplementary Figure S7.1(b)** illustrates the overall structure of the MGM and the generation process of the target point cloud, also known as the reverse diffusion process. The MGM contains three components: a multimodal foundation model for extracting multimodal semantic embedding $\alpha$ from multimodal prompts (texts and point clouds in this experiment); a denoising U-Net^[21]^ used to predict the noise that should be removed at each step of the reverse diffusion process, based on multimodal semantic embedding $\alpha$; an autoencoder that maps the point cloud $x$ to the latent variable $z$. Specifically, the noise is removed during the reverse diffusion process according to the following equation:

$z_{t-1}=\frac{1}{\sqrt{\eta_{t}}}\left( z_{t}-\frac{1-\eta_{t}}{\sqrt{1-\bar{\eta}_{t}}}\varepsilon_{\theta,t} \right)+\sqrt{\frac{1-\bar{\eta}_{t-1}}{1-\bar{\eta}_{t}}\beta_{t}}\varepsilon.$ (7)

where $\eta_{t}=1-\beta_{t}$, $\bar{\eta}_{t}=\prod_{i=1}^{t} \eta_{i}$, $\varepsilon_{\theta,t}=\varepsilon_{\theta}(z_{t},t,\alpha)$ is the noise predicted by the denoising U-Net at step $t$ based on multimodal semantic embedding $\alpha$ and latent variable $z_{t}$, $\theta$ is the parameter of the denoising U-Net, and $\varepsilon$ is a random variable sampled from the standard Gaussian distribution. **Supplementary Figure S7.1(c)** shows the structure of the autoencoder, consisting of two shared MLPs, which share weights between points. The input to the encoder is the point cloud $x$, which, after processing through the shared MLP and global max pooling, is transformed into the 1D latent variable $z$. This $z$ is duplicated and combined with a randomly sampled point cloud $C_{init}$ to form the input to the decoder, which outputs the reconstructed point cloud $\hat{x}$ via the shared MLP. **Supplementary Figure S7.1(d)** describes the structure of the denoising U-Net, which includes three Down Blocks, one Middle Blocks, three Up Blocks, and an output layer (Conv$1\times1$). The input feature map is a combination of the latent variable $z_{t}$, time embedding $t_{emb}$ that includes timestep information, and the multimodal semantic embedding $\alpha$ extracted from the multimodal prompt by the multimodal foundation model. The time embedding $t_{emb}$ is formed by parameters [$\beta_{t}$, $sin(\beta_{t})$, $cos(\beta_{t})$]; $t_{emb}$ needs to be replicated before it can be concatenated with the other two feature maps. Each Down Block halves the dimension of the feature map while doubling the number of channels, and each Up Block doubles the dimension while halving the number of channels. The output of the denoising U-Net is the noise that should be removed at step $t$. **Supplementary Figure S7.1(e)** shows the structure of the multimodal foundation model, which is composed of a frozen pre-trained CLIP model, shared MLP, and MLPs. The text prompt is converted into the original text embedding by the CLIP model, then compressed into the text embedding by the MLP; the point cloud prompt is processed by the shared MLP and the point cloud embedding is obtained after global max pooling and MLP. These two embeddings are concatenated to form the multimodal semantic embedding $\alpha$. **Supplementary Figure S7.1(f)** shows the Downs Block, the Middle Block and the Up block used in denoising U-Net.

We want to be able to control the importance of guidance of multimodal semantic embeddings on the generation process in a finer way, so we use a technique called classifier-free guidance (CFG) ^[22]^, which expresses the diffusion model's weighting of the importance of conditional information by introducing a hyperparameter:

$\nabla\log p(z_{t}|\alpha)=\nabla\log p\left( z_{t} \right)+w\nabla\log p\left( \alpha|z_{t} \right).$ (8)

(8) implicitly introduce a classifier $p\left( \alpha|z_{t} \right)$ that is up-weighted by assigning high likelihood on-data probabilities to the correct labels. By Bayes' law $p\left( \alpha|z_{t} \right)\propto p\left( z_{t}|\alpha\right)/p(z_{t})$, and assuming $\nabla\log p\left( z_{t}|\alpha\right)=-\frac{1}{\sigma_{t}}(\varepsilon_{\theta}(z_{t},t,\alpha))$, $\nabla\log p\left( z_{t} \right)=-\frac{1}{\sigma_{t}}(\varepsilon_{\theta}(z_{t},t))$, where $\sigma_{t}=\sqrt{\frac{1-\bar{\eta}_{t-1}}{1-\bar{\eta}_{t}}\beta_{t}}$. So, the above equation can be transformed into:

$\bar{\varepsilon}_{\theta}(z_{t},t,\alpha)=\left( 1-w \right)\varepsilon_{\theta}\left( z_{t},t \right)+w\varepsilon_{\theta}\left( z_{t},t,\alpha\right).$ (9)

Here, when $w=0$, the conditional information does not work because there is no classifier gradient, and when $w>0$, the larger $w$ is, the stronger the guidance from the conditional information is. So, we can fit $\varepsilon_{\theta}\left( z_{t},t,\alpha\right)$ and $\varepsilon_{\theta}\left( z_{t},t \right)$ by training two diffusion models, a conditional diffusion model and an unconditional diffusion model. However, in practice, we generally perform a random dropout on the conditional information, e.g., replacing the text with an empty string, so that only one diffusion model needs to be trained.

The training of the entire MGM is divided into two phases. First is the training of the autoencoder, we train the autoencoder using the following loss function:

$L_{A}=\frac{1}{M}\sum_{i=1}^{M} \{\left[ \frac{1}{\left| x_{i} \right|}\sum_{c\in x_{i}} \min_{\hat{c}\in\hat{x}_{i}} \left\| c-\hat{c} \right\|^{2} \right]+\left[ \frac{1}{\left| \hat{x}_{i} \right|}\sum_{\hat{c}\in\hat{x}_{i}} \min_{c\in x_{i}} \left\| \hat{c}-c \right\|^{2} \right]\}.$ (10)

where $M$ is the total number of training data, $x_{i}$ is the $i$-th point cloud in the training dataset, $\hat{x}_{i}$ is the point cloud reconstructed by the autoencoder, $c$ is the point in $x_{i}$, and $\hat{c}$ is the point in $\hat{x}_{i}$. After training the autoencoder, all training samples are mapped to the latent space to form a dataset for training the diffusion model and the multimodal foundation model. During the training of the diffusion model and the multimodal foundation model, we mask the prompt with a probability by substituting the original text and point cloud prompts with empty strings and zero point clouds, respectively. The loss function for the diffusion model is defined as:

$L_{\theta}=E_{t,z_{t},\varepsilon,\alpha}[\left\| \varepsilon_{\theta}\left( z_{t},t,\alpha\right)-\varepsilon\right\|_{2}^{2}].$ (11)

After training the diffusion model, the strength of prompt guidance can be adjusted by the (9).

In this experiment, point cloud prompts are obtained by randomly sampling on the real point clouds. The point cloud of target contains 2048 points, and the dimension of the latent variable $z$ is set to $32\times32=1024$. The CLIP model, serving as a pre-trained model, remains frozen throughout the training process, with its original text embedding dimension being 768. The dimensions of the text embedding are $32\times32\times1$, and those of the point cloud embedding are $32\times32\times1$. The autoencoder is trained using the Adam optimizer, with $\beta_{1}$ set at 0.9 and $\beta_{2}$ at 0.99. The batch size is set to 64, and the learning rate is 0.001, with the training spanning 3000 epochs. In the training of the diffusion model, the probability of masking the prompt is set at 0.1, with a total of 1000 diffusion steps. The variance hyperparameters $\beta_{1}$ and $\beta_{T}$ are set to 0.0001 and 0.02, respectively, adjusted using a linear scheduler. The optimizer remains Adam, with $\beta_{1}$ at 0.9 and $\beta_{2}$ at 0.99. The batch size is 128, the learning rate is 0.001, and the training encompasses a total of 10000 epochs.

**Supplementary Notes S8. Details of Physical Adapter in the 3D Microwave Meta-imaging Experiment**

**
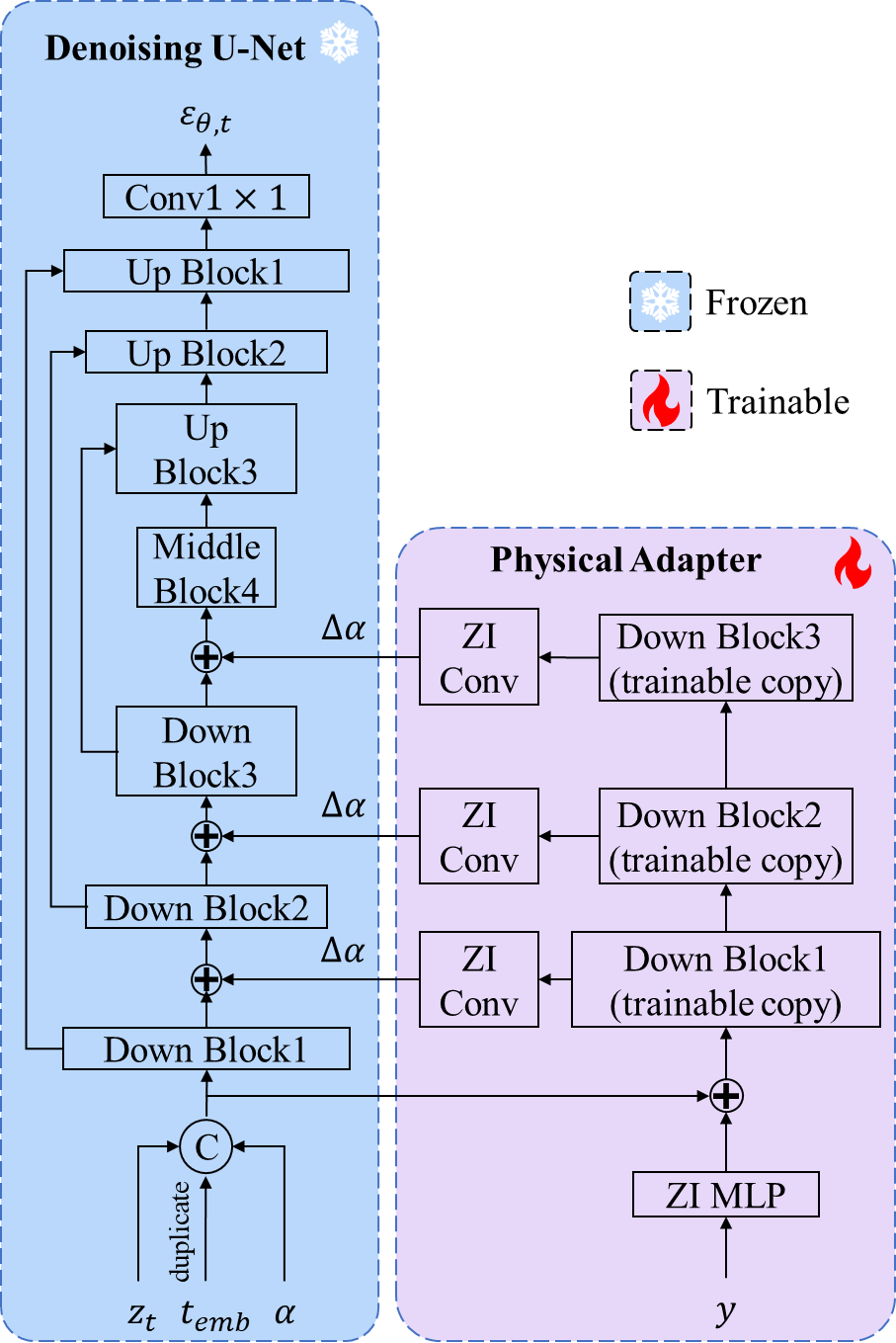
**

**Supplementary Figure S8.1** | **The physical adapter used in 3D Microwave Meta-imaging Experiment.** Here, ‘ZI’ means zero initialization; ‘trainable copy’ means that the parameters initialized at the beginning of training are those of the original module; ‘Down Block’ as shown in **Supplementary Figure S7.1(f)**.

**Supplementary Figure S8.1** shows the physical adapter used in 3D microwave meta-imaging. The physical adapter includes multiple "trainable copies" of the down block and "zero-initialized" convolution layers. The input, physical measurements $y$, are dimensionally matched through a "zero-initialized" MLP before being combined with the input of the MGM, which is obtained by concatenating the hidden variable $z$, the time embedding $t_{emb}$ and the multimodal semantic embedding $\alpha$. It then passes through multiple "trainable copies" and "zero-initialized" convolution layers, ultimately inputting the residual low-level parameter $\Delta\alpha$ into the MGM. During training, the physical adapter is trained using collected paired data, with the parameters of the MGM frozen and multimodal prompts probabilistically masked to enhance the physical adapter's capability to extract information from physical measurements $y$, serving as a substitute for multimodal prompts. The training loss is defined as:

$L_{\varphi}=E_{x,\alpha,z,y}[\left\| x-\mathcal{G}(\alpha,\mathcal{M}_{\varphi}(y),z) \right\|_{2}^{2}].$ (12)

In the experiments, the physical adapter includes three "trainable copies," three "zero-initialized" convolution layers with a kernel size of 1, and one "zero-initialized" MLP. The masking probability for prompts is set at 0.1, batch size at 512, learning rate at 0.0001, using the Adam optimizer with $\beta_{1}$ at 0.9 and $\beta_{2}$ at 0.99, over a total of 3000 training epochs.

**Supplementary Notes S9. Supplementary Results on 3D Microwave Meta-imaging for 5.5 GHz Systems**

**
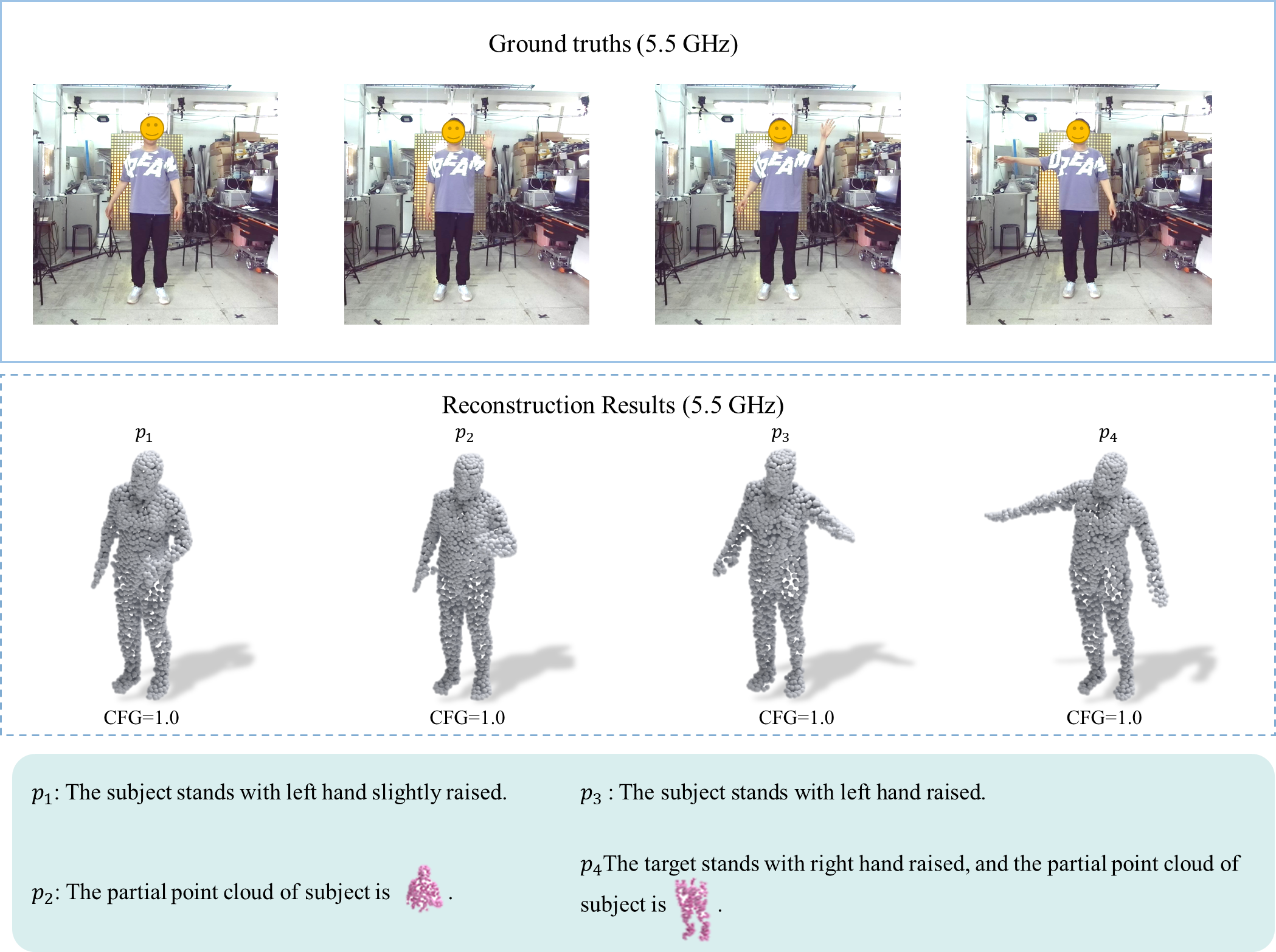
**

**Supplementary Figure S9.1 | Supplementary results on 3D microwave meta-imaging for 5.5 GHz systems.**

**Supplementary Figure S9.1** shows some imaging results of the 3D microwave meta-imaging on the 5.5GHz system. In this experiment, we retained the original MGM and only retrained the physical adapter. The results indicate that, visually, the imaging accuracy of our framework on the 5.5GHz system is almost identical to that on the 2.4GHz system.

**Supplementary Notes S10. Supplementary Results with Comparison Experiment with End-to-end Network**

**
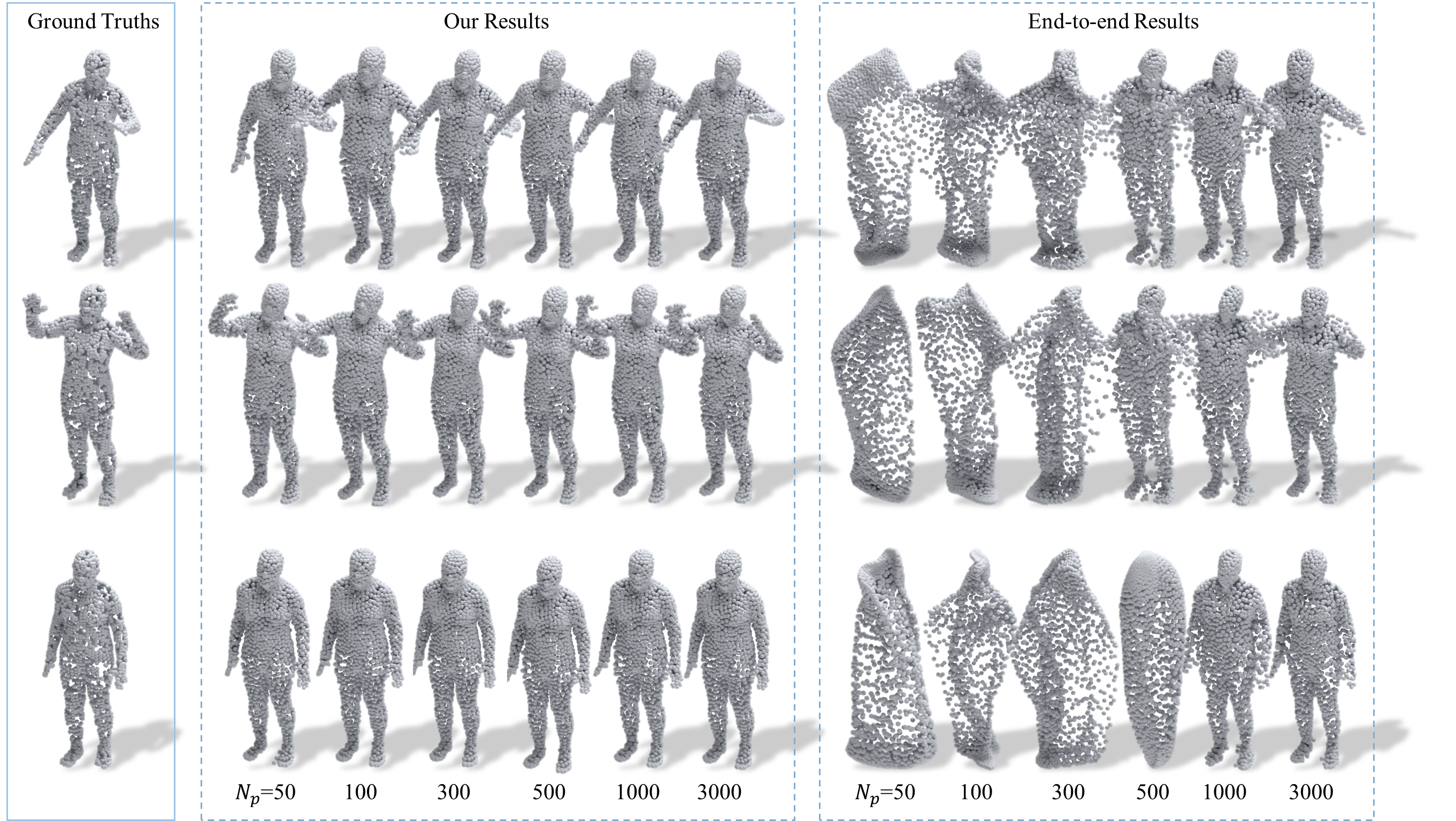
**

**Supplementary Figure S10.1 | Supplementary results with comparison experiment with end-to-end network with different number of the paired training data.**

**Supplementary Figure S10.1** shows the imaging results of our method with end-to-end network trained with different amounts of training data. To ensure fairness, we did not use multimodal priors for imaging. These imaging results show that with limited training data (less than 300 samples), the end-to-end network produces poor imaging results, often failing to form a human shape; only when the training data reaches 1,000 does the end-to-end network manage to achieve marginally acceptable imaging results. In contrast, our framework can still produce good imaging results even when the training data is as low as 50 samples. Although there may be errors in the target's posture, the imaging consistently maintains a good human form. This is thanks to the MGM, which, during training, learns the common sense and features related to human posture. This allows the physical adapter to focus solely on mapping the observational data to human posture features, without having to construct the posture features from scratch—this part is handled by the MGM. Meanwhile, the end-to-end network has to learn both the mapping from observational data to human posture features and construct the posture features itself. With insufficient training data, the end-to-end network struggles to accurately build human posture features, resulting in imaging that often lacks a human form.

**Supplementary Notes S11. Details of 4D Compressive Microwave Meta-imaging Experiment**

**Visual-semantic map.**


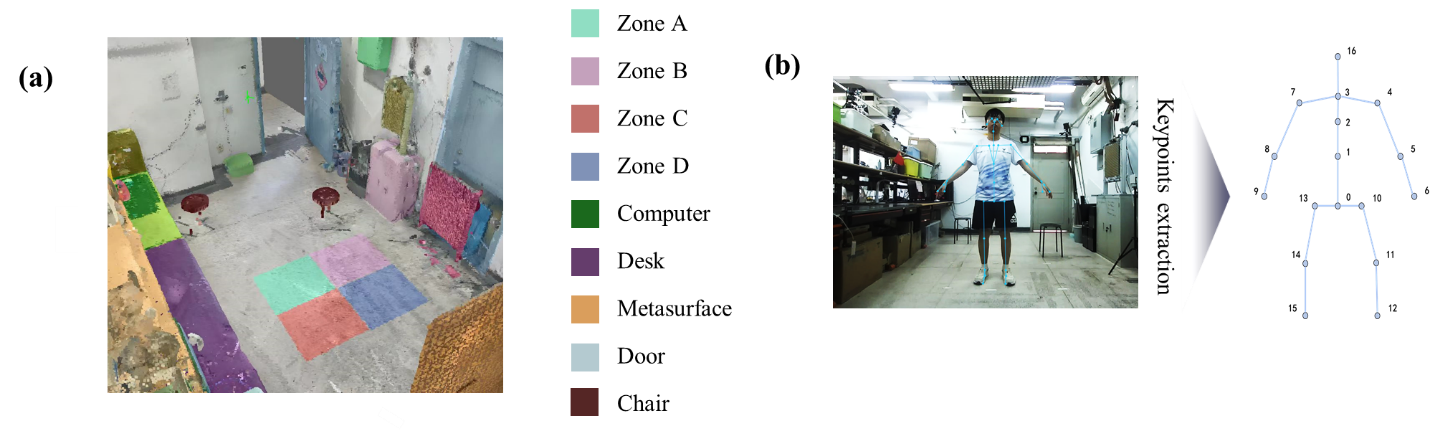


**Supplementary Figure S11.1** | **The setup of 4D compressive microwave meta-imaging experiment. (a)** The visual-semantic map of the laboratory environment. **(b)** The extraction process of skeleton keypoints, where 34 main skeleton points are automatically extracted from the photos taken by ZED2 after being processed by its built-in SDK, and the most critical 17 skeleton points are selected as labels.

Here, we provide a introduction to the development of a 3D visual-semantic map, specifically tailored to address the complex challenges of 4D compressive microwave meta-imaging. To construct this map, we first scan the laboratory with a ZED2 camera, then convert the scanned data into either 3D point clouds using the SDK integrated within the ZED2 system. Following this, in a meticulous process, we manually annotate different areas of the lab with semantic labels such as ‘computer’, ‘chair’, and ‘cabinet’, and categorize and label the floor areas. This effort culminates in the creation of an exhaustive semantic map, similar to the one depicted in **Supplementary Figure S11.1(a)**.

Thus, for any given set of coordinates, we are able to precisely translate them into specific semantic locations on the map, surpassing the simple numerical data provided by the ZED2. For example, the coordinates (1.1m, 2.1m, 1.5m) are interpreted as "in front of the desk", while (1.3m, 1.2m, 0.8m) indicates "on the left side of the chair". This intuitive and precise description based on the semantic map greatly enriches the process of creating and annotating the dataset, enabling us to depict human behavior more vividly. The remaining system configurations for the experiment continue as outlined in the previous section.

**Dataset collection and preparation.**

In our 4D compressive microwave meta-imaging experiments conducted under 2.4GHz and 5.0GHz systems, we use 3D skeleton sequences to describe the targets. For supervision purposes, we need to align optical data with microwave data, during which we precisely extract a 34-key-point 3D skeleton from the original optical images using the SDK integrated into the ZED2 camera. Considering the need to balance imaging speed and accuracy, we have simplified these key points to 17, covering the head, trunk, limbs, and other important body areas, as shown in **Supplementary Figure S11.1(b)**. We then compile this continuous skeleton data into video format and annotate it semantically to construct our 4D dataset. In our experimental setup, we define every 20 frames as a complete 4D sequence.

Additionally, to reduce jitter and outliers in the training dataset, we employ temporal domain filtering and smoothing techniques. We organize the continuous microwave measurement data into sequences, maintaining consistency with the 20-frame format of the skeleton data. These measures ensure the accuracy of the experimental data and the efficiency of the experimental process.

**Supplementary Notes S12. Details of MGM and Training Algorithm in the 4D Microwave Meta-imaging Experiment**

**
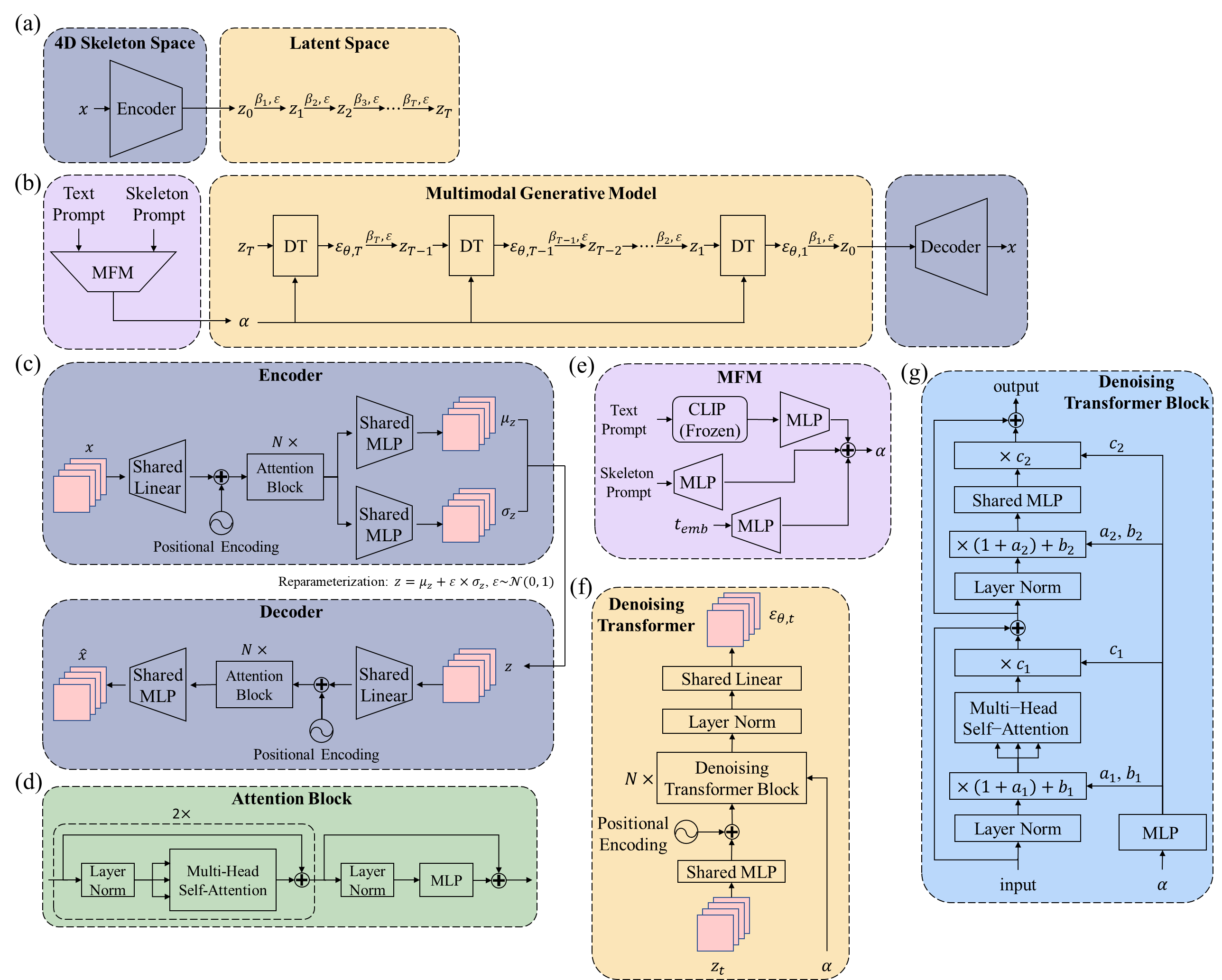
**

**Supplementary Figure S12.1** | **Internal composition of the MGM for 4D microwave meta-imaging.** **(a)** Diffusion processes of the diffusion model. **(b)** Overall structure of the MGM and the reverse diffusion process of the diffusion model. **(c)** The architecture of the ‘Variational Autoencoder’. **(d)** The architecture of the ‘Attention Block’. **(e)** The architecture of the ‘Multimodal Foundation Model’. **(f)** The architecture of the ‘Denoising Transformer’. **(g)** The architecture of the ‘Denoising Transformer Block’. Here, ‘MFM’ stands for the Multimodal Foundation Model; ‘DT’ is the Denoising Transformer; ‘Shared' indicates that the module shares parameters at the sequence time step, ‘Linear’ represents a linear fully connected layer; ‘Layer Norm’ means the layer normalization.

In the 4D microwave meta-imaging experiment, we continue to use the diffusion model as the MGM. The specific network structure is shown in **Supplementary Figure S12.1**. **Supplementary Figure S12.1(a)** illustrates the diffusion process of the diffusion model. As in previous experiments, we still map 4D skeleton data to the latent space and generate it within this space. **Supplementary Figure S12.1(b)** illustrates the overall structure of the MGM and the generation process of the target, also known as the reverse diffusion process. Therefore, the MGM includes three modules: a multimodal foundation model for extracting multimodal semantic embedding $\alpha$ from multimodal prompts (text and first frame in this experiment); a variational autoencoder (VAE)^[23]^ that maps the 4D skeleton to the latent space; a denoising Transformer^[24]^ used to predict the noise that should be removed at each step of the reverse diffusion process, based on multimodal semantic embedding $\alpha$. **Supplementary Figure S12.1(c)** shows the structure of the VAE. The encoder includes a shared linear layer, two shared MLPs, and multiple Attention Blocks. Its input is the 4D skeleton, and it outputs the mean and standard deviation of the latent variables. The data first passes through a linear layer that shares parameters across time steps for dimensional transformation, then after adding positional encoding, it goes through several concatenated Attention Blocks for feature extraction. The extracted features are processed by two shared MLPs to determine the distribution of the latent variables $\mathcal{N(}\mu_{z},\sigma_{z}^{2})$. The structure of the decoder is similar to that of the encoder, including a shared linear layer, a shared MLP, and multiple Attention Blocks. First, the latent variable $z$ is sampled from the distribution of latent variables obtained from the encoder using the reparameterization trick. Specifically, a variable $\varepsilon$ is sampled from a standard normal distribution, and then the latent variable $z$ is calculated using $z=\mu_{z}+\varepsilon\times\sigma_{2}$. This allows the gradient to be backpropagated during training. After processing the latent variable $z$ through the shared linear layer and adding positional encoding, it passes through several Attention Blocks and a shared MLP to reconstruct the 4D skeleton. **Supplementary Figure S12.1(d)** shows the structure of the Attention Block, which includes two residual multi-head self-attention layers and MLP, with Layer Norm for normalization. **Supplementary Figure S12.1(e)** shows the structure of the multimodal foundation model, which is composed of a frozen pre-trained CLIP model and MLPs. The text prompt is converted into the original text embedding by the CLIP model, then compressed into the text embedding by the MLP; the skeleton prompt is processed by the MLP and the skeleton embedding is obtained. The time embedding $t_{emb}$ is processed by the MLP and the compressive time embedding is obtained. These three embeddings are added to form the multimodal semantic embedding $\alpha$. **Supplementary Figure S12.1(f)** presents the structure of the denoising Transformer, which consists of multiple denoising Transformer Blocks, MLP, and shared linear layer. The latent variable $z_{t}$ is processed by a shared MLP and, after being added to positional encoding, is fed into multiple denoising Transformer blocks. At the same time, multimodal semantic embedding $\alpha$ is also input into each denoising Transformer block. After being processed by multiple denoising Transformer blocks, the data undergoes layer normalization and passes through a shared linear layer, ultimately outputting the predicted noise $\varepsilon_{\theta,t}=\varepsilon_{\theta}(z_{t},t,\alpha)$ that needs to be removed. **Supplementary Figure S12.1(g)** displays the structure of the denoising Transformer Block, which includes MLP, multi-head self-attention, and Layer Norm. The input is sequentially processed through a residual multi-head self-attention and a residual MLP to obtain the output. The condition containing prompt information is transformed into six parameters $(a_{1},b_{1},c_{1},a_{2},b_{2},c_{2})$ via an MLP, which are used to adjust the scaling and shifting of features, thereby controlling the model behavior to generate the desired output.

The training of the MGM is divided into two stages. First, we train the VAE and optimize it according to the following loss function:

$L_{V}=\frac{1}{2M}\sum_{i=1}^{M} [\left\| x_{i}-\hat{x}_{i} \right\|_{2}^{2}+\lambda\left( -\log\sigma_{zi}^{2}+\mu_{zi}^{2}+\sigma_{zi}^{2}-1 \right)].$ (13)

where $M$ is the total number of training data, $x_{i}$ is the $i$-th training data in the training dataset, $\hat{x}_{i}$ is the target reconstructed by the VAE. The first term is the reconstruction loss, the second term is the Kullback-Leibler (KL) loss, and $\lambda$ is the weight of the KL loss. $\mu_{i}$ and $\sigma_{i}$ are the mean and standard deviation of the latent variable distribution of the $i$-th training sample output by the encoder. After the VAE training is complete, we map all training samples to the latent space to create a training set of latent variables, which is used to train the diffusion model. The training of the diffusion model also employs a CFG strategy. The loss function of the diffusion model is defined as shown in (11).

In this experiment, the dimension of the latent variable $z$ is 20$\times$64, and the dimension of the text embedding is 768. In the VAE, both the encoder and decoder contain three Attention Blocks, each with four heads and 256 hidden units. The training of the VAE uses the Adam optimizer, with $\beta_{1}$ set to 0.9 and $\beta_{2}$ set to 0.99, and $\lambda$ in (6) set to 1e-6. The batch size is set to 64, the learning rate is 0.0001, and the total number of training epochs is 5000. In the training of the diffusion model, the number of denoising Transformer Blocks is 12, each block containing four heads and 256 hidden units. During training, the text prompt masking probability is set to 0.1, and the number of diffusion steps is 50. The variance hyperparameters $\beta_{1}$ are set to 0.0001, and $\beta_{T}$ to 0.02, adjusted using a linear scheduler. The optimizer remains Adam, with $\beta_{1}$ at 0.9 and $\beta_{2}$ at 0.99. The batch size is set to 256, the learning rate is 0.0001, and the total number of training epochs is 10000.

**Supplementary Notes S13. Details of Physical Adapter in the 4D Microwave Meta-imaging Experiment**

**
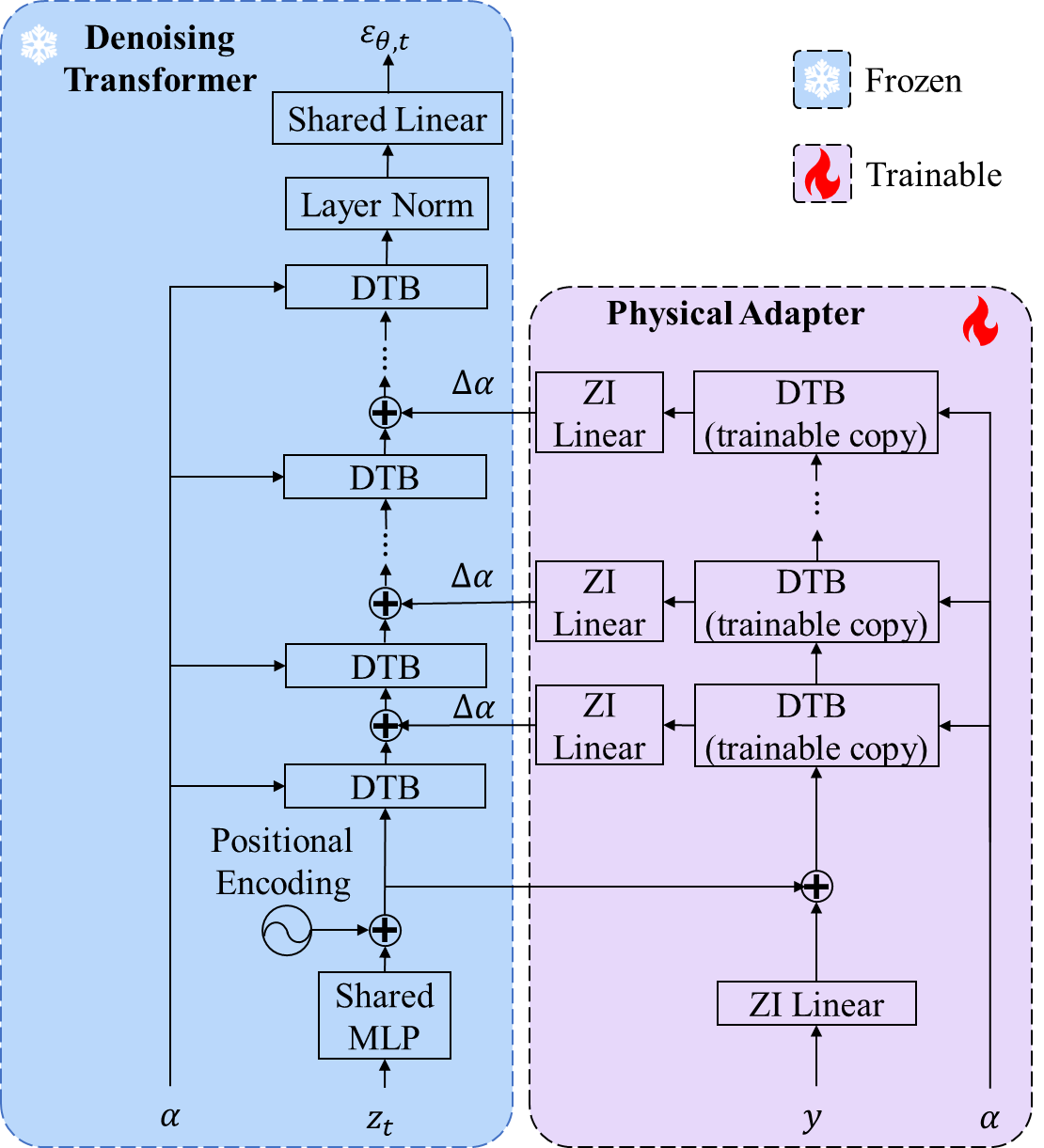
**

**Supplementary Figure S13.1** | **The physical adapter used in 4D Microwave Meta-imaging Experiment.** Here, ‘ZI’ means zero initialization; ‘trainable copy’ means that the parameters initialized at the beginning of training are those of the original module; ‘DTB’ is the Denoising Transformer Block, as shown in **Supplementary Figure S12.1(g)**.

**Supplementary Figure S13.1(c)** illustrates the physical adapter used in 4D microwave meta-imaging. The physical adapter includes multiple "trainable copies" of the denoising Transformer block (DTB) and "zero-initialized" linear layers. The input, physical observations, is dimensionally matched through a "zero-initialized" linear layer before being combined with the input of the MGM, processed through multiple "trainable copies" and "zero-initialized" linear layers, and ultimately outputting the residual low-level parameter $\Delta\alpha$ into the backbone network, where the "trainable copies" of the DTB are also guided by the multimodal semantic embedding $\alpha$. During training, MGM’s parameters are frozen, and prompts are probabilistically masked. The training loss is defined as (12). In the experiments, the physical adapter includes three "trainable copies" of the DTB, with a prompt masking probability of 0.1, batch size set at 512, learning rate at 0.0001, using the Adam optimizer with $\beta_{1}$ at 0.9 and $\beta_{2}$ at 0.99, over a total of 3000 training epochs.

**Supplementary Notes S14. Supplementary Results on the Effects of the Importance of Semantic Prior on Performance in 4D Meta-imaging**

**
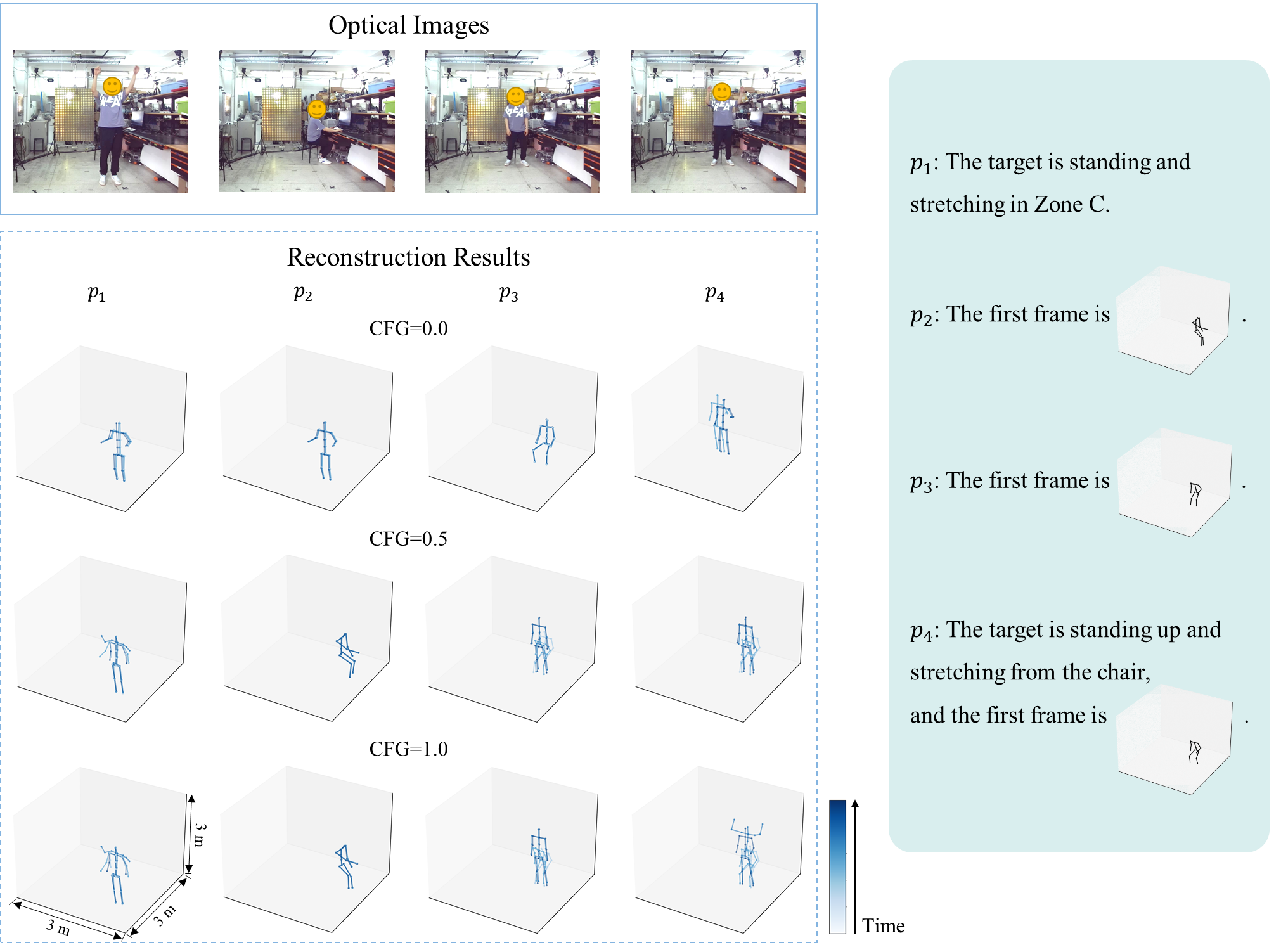
**

**Supplementary Figure S14.1 | Supplementary results on the effects of the importance of semantic prior on performance in 4D meta-imaging.**

**Supplementary Figure S14.1** shows the selected results on the effects of the importance of semantic prior on performance in 4D meta-imaging. We can observe that the imaging results become more precise with the amount of prior information increases. Since the initial frame priors of $p_{4}$ and $p_{3}$ are the same, when CFG is set at 0.5, the guiding strength of the prior is minimal, making the text prior in $p_{4}$ ineffective and resulting in an imaging outcome nearly identical to that of $p_{4}$, where the target is merely standing up from the chair. However, when CFG is increased to 1.0, the text prior becomes effective, leading to a more precise imaging result, showing the target stretching. Now, it can be observed from these results that, consistent with previous experiments, the prior is capable of compensating for the inadequate and noisy measurements, thereby improving imaging accuracy.

**References**

[1] LeCun, Y., Bottou, L., Bengio, Y., & Haffner, P. Gradient-based learning applied to document recognition.*Proceedings of the IEEE* **86**, 2278-2324 (1998).

[2] Collins, J. D., Volakis, J. L., & Jin, J. M. A combined finite element-boundary integral formulation for solution of two-dimensional scattering problems via CGFFT. *IEEE transactions on antennas and propagation* **38**, 1852-1858 (1990).

[3] Goodfellow, I., Pouget-Abadie, J., Mirza, M., Xu, B., Warde-Farley, D., Ozair, S., ... & Bengio, Y. Generative adversarial networks. *Communications of the ACM* **63**, 139-144 (2020).

[4] Radford, A., Kim, J. W., Hallacy, C., Ramesh, A., Goh, G., Agarwal, S., ... & Sutskever, I. Learning transferable visual models from natural language supervision. *International conference on machine learning* (2021).

[5] Tolstikhin, I. O. et al. Mlp-mixer: An all-mlp architecture for vision.  *Advances in neural information processing systems* (2021).

[6] Podell, D., English, Z., Lacey, K., Blattmann, A., Dockhorn, T., Müller, J., ... & Rombach, R. Sdxl: Improving latent diffusion models for high-resolution image synthesis. *arXiv preprint* (2023).

[7] Thomee, B., Shamma, D. A., Friedland, G., Elizalde, B., Ni, K., Poland, D., Borth, D., & Li, L.-J. Yfcc100m: The new data in multimedia research. *Communications of the ACM* (2016).

[8] Krizhevsky, A., Sutskever, I., & Hinton, G. E. Imagenet classification with deep convolutional neural networks. *Advances in neural information processing systems* (2012).

[9] Xu, B., Wang, N., Chen, T., & Li, M. Empirical evaluation of rectified activations in convolutional network. *arXiv preprint* (2015).

[10] Gulrajani, I., Ahmed, F., Arjovsky, M., Dumoulin, V., & Courville, A. C. Improved training of wasserstein gans. *Advances in neural information processing systems* (2017).

[11] Xie, S., & Tu, Z. Holistically-nested edge detection. *Proceedings of the IEEE international conference on computer vision* (2015).

[12] Zhang, L., Rao, A., & Agrawala, M. Adding conditional control to text-to-image diffusion models. *Proceedings of the IEEE/CVF International Conference on Computer Vision* (2023).

[13] Steiner, B., DeVito, Z., Chintala, S., Gross, S., Paske, A., Massa, F., ... & Bai, J. Pytorch: An imperative style, high-performance deep learning library. (2019).

[14] Kingma, D. P., & Ba, J. Adam: A method for stochastic optimization. *arXiv preprint* (2014).

[15] Ulyanov, D., Vedaldi, A., & Lempitsky, V. Deep image prior, *Proceedings of the IEEE conference on computer vision and pattern recognition* (2018).

[16] Mataev, G., Milanfar, P., & Elad, M. DeepRED: Deep image prior powered by RED.*Proceedings of the IEEE/CVF International Conference on Computer Vision Workshops* (2019).

[17] Wang, F., Bian, Y., Wang, H., Lyu, M., Pedrini, G., Osten, W., ... & Situ, G. Phase imaging with an untrained neural network.*Light: Science & Applications* **9**, 77 (2020).

[18] Wang, Y. M., & Chew, W. C. An iterative solution of the two‐dimensional electromagnetic inverse scattering problem.*International journal of imaging systems and technology* **1**, 100-108 (1989).

[19] Pavlakos, G., Choutas, V., Ghorbani, N., Bolkart, T., Osman, A. A., Tzionas, D., & Black, M. J. Expressive body capture: 3d hands, face, and body from a single image, *Proceedings of the IEEE/CVF conference on computer vision and pattern recognition* (2019).

[20] Ho, J., Jain, A., & Abbeel, P. Denoising diffusion probabilistic models. *Advances in neural information processing systems* **33**, 6840-6851 (2020).

[21] Ronneberger, O., Fischer, P., & Brox, T. U-net: Convolutional networks for biomedical image segmentation. *Medical Image Computing and Computer-Assisted Intervention–MICCAI 2015: 18th International Conference* (2015).

[22] Ho, J., & Salimans, T. Classifier-free diffusion guidance. *arXiv preprint* (2022).

[23] Kingma, D. P., & Welling, M. Auto-encoding variational bayes. *arXiv preprint* (2013).

[24] Peebles, W., & Xie, S. Scalable diffusion models with transformers. Proceedings of the IEEE/CVF International Conference on Computer Vision (2023).
